# Commensal-Related Changes in the Epidermal Barrier Function Lead to Alterations in the Benzo[*a*]Pyrene Metabolite Profile and Its Distribution in 3D Skin

**DOI:** 10.1128/mBio.01223-21

**Published:** 2021-09-28

**Authors:** Lisa Lemoine, Dilan Bayrambey, Alexander Roloff, Christoph Hutzler, Andreas Luch, Tewes Tralau

**Affiliations:** a German Federal Institute for Risk Assessment, Department of Pesticides Safety, Berlin, Germany; b Institute of Pharmacy, Department of Biology, Chemistry, Pharmacy, Freie Universität Berlin, Berlin, Germany; c German Federal Institute for Risk Assessment, Department of Chemical & Product Safety, Berlin, Germany; The Jackson Laboratory; Georgia Institute of Technology School of Biological Sciences

**Keywords:** coculture, skin model, commensals, benzo[*a*]pyrene, metabolites, skin barrier, GC-MS, BPDE DNA adducts, epidermal barrier

## Abstract

Polycyclic aromatic hydrocarbons (PAH) such as benzo[*a*]pyrene (B[*a*]P) are among the most abundant environmental pollutants, resulting in continuous exposure of human skin and its microbiota. However, effects of the latter on B[*a*]P toxicity, absorption, metabolism, and distribution in humans remain unclear. Here, we demonstrate that the skin microbiota does metabolize B[*a*]P on and in human skin *in situ*, using a recently developed commensal skin model. In this model, microbial metabolism leads to high concentrations of known microbial B[*a*]P metabolites on the surface as well as in the epidermal layers. In contrast to what was observed for uncolonized skin, B[*a*]P and its metabolites were subject to altered rates of skin penetration and diffusion, resulting in up to 58% reduction of metabolites recovered from basal culture medium. The results indicate the reason for this altered behavior to be a microbially induced strengthening of the epidermal barrier. Concomitantly, colonized models showed decreased formation and penetration of the ultimate carcinogen B[*a*]P-7,8-dihydrodiol-9,10-epoxide (BPDE), leading, in consequence, to fewer BPDE-DNA adducts being formed. Befittingly, transcript and expression levels of key proteins for repairing environmentally induced DNA damage such as xeroderma pigmentosum complementation group C (XPC) were also found to be reduced in the commensal models, as was expression of B[*a*]P-associated cytochrome P450-dependent monooxygenases (CYPs). The results show that the microbiome can have significant effects on the toxicology of external chemical impacts. The respective effects rely on a complex interplay between microbial and host metabolism and microbe-host interactions, all of which cannot be adequately assessed using single-system studies.

## INTRODUCTION

The skin is one of our largest organs and, as a physical and metabolic barrier, is our front line of defense against environmental impacts, physical as well as chemical (1). Moreover, it harbors a unique ecosystem of 10 to 100 trillion bacteria, fungi and viruses, which together make up the skin microbiota (2–4). As a community with a complex interplay, this microbiota relies to a large extent on the few nutrients it can obtain from the stratum corneum (SC) as well as sebaceous, eccrine, and apocrine secretions (5). However, with the skin being a chronically nutrient-poor environment, the microbial commensals have to rely on the use of external carbon and energy sources in the form of xenobiotics (6, 7). Given the preferred growth of commensals in and on the SC, major xenobiotic sources of additional nutrients comprise reoccurring environmental exposures, from airborne pollutants to cosmetic ingredients or topically applied pharmaceuticals (8).

There is abundant evidence that the human microbiome can modulate exposure-response relationships of xenobiotic chemicals through some general mechanisms that could directly or indirectly affect toxicity (9). Respective mechanisms include direct metabolic conversions as well as indirect transformations. The latter include deconjugation of host-generated metabolites, modulation of epithelial barrier permeability, and regulation or alteration of endogenous host metabolism (9–14).

Among the most widespread environmental xenobiotics are polycyclic aromatic hydrocarbons (PAHs). Their ubiquitous occurrence leads to constant human exposure at low to medium levels (15, 16). Of particular toxicological interest are the potentially carcinogenic, high-molecular-weight representatives such as benzo[*a*]pyrene (B[*a*]P) (17, 18). With regard to the latter, it is actually the oxidative metabolic activation by cytochrome P450-dependent monooxygenases (CYPs) during human phase I metabolism that can trigger the formation of some of the most potent genotoxic carcinogens (19). For the metabolic activation of B[*a*]P, two of the most important enzymes involved in the formation of the ultimately reactive species are CYP1A1 and CYP1B1 (20). The resulting electrophilic metabolite B[*a*]P-7,8-dihydrodiol-9,10-epoxide (BPDE) is prone to DNA adduct formation, preferentially at nucleophilic guanine residues, leading to the formation of dG-N2-BPDE (21). While the formation of the biologically dysfunctional dG-N2-BPDE makes BPDE a strong genotoxin, overall toxicity will strongly depend on any phase I and phase II detoxification preceding actual adduct formation as well as on the rate of DNA repair (22). It should be noted, however, that before being subject to host metabolism, PAHs obviously first need to pass the epidermis, which is a highly functional physicochemical barrier (23).

Exposure to PAHs predominantly occurs first on the skin with human commensal microbes hence being the first point of (metabolic) contact (24, 25). Previous studies from our lab have shown a ubiquitous potential of the commensal microbiome for B[*a*]P-degradation. Some of the bacterial genes for the respective underlying metabolic pathways were identified and shown to be detectable *in situ* on human skin, as were the associated bacteria. Concomitantly carbon-limited batch cultures showed the corresponding bacterial metabolism of B[*a*]P to result in the formation and excretion of highly cytotoxic and genotoxic metabolites (26, 27). Similar observations have been reported for the intestinal microbiota, where commensals are known to directly metabolize xenobiotic substances, as well as impacting the host’s capacity for xenobiotic metabolism by affecting phase I and phase II enzymes (28–30).

While these studies deliver valuable insights into the various aspects of how commensal metabolism of xenobiotics can influence host toxicity, they fall short of addressing the complex and faceted interplay systemically. The reason for this is commensal species specificity together with a lack of suitable model systems. We therefore recently reported the development of a new commensal 3D skin model (31). We have now applied this model to investigate commensal B[*a*]P-metabolism and its potential toxicologically relevant effects *in situ*. These results provide insights into the complex interplay between microbially induced xenobiotic metabolism of B[*a*]P as a procarcinogen and the host organ skin under near *in vivo* conditions (see Data Set S1 for experimental setup). The data indicate a complex pattern of host-microbe interactions that were not accessible in previous single-system studies.

10.1128/mBio.01223-21.1 DATA SET S1 Experimental setup and study design. A surface imprint for bacterial quantification as well as for chemical analysis and the examination of the skin models themselves took place on day 8 of cultivation. Sampling of the medium was done daily. Download Data Set S1, JPG file, 2.6 MB. Copyright © 2021 Lemoine et al. 2021 Lemoine et al. https://creativecommons.org/licenses/by/4.0/ This content is distributed under the terms of the Creative Commons Attribution 4.0 International license.

## RESULTS

The aim of the study was to investigate the effects of commensal skin colonization on B[*a*]P-metabolism *in situ* using a microbially competent 3D skin model (31). The model was colonized with two previously isolated skin commensals, namely, Micrococcus luteus 1B and Pseudomonas oleovorans 1C (26, 27). While both organisms have been established using B[*a*]P as the sole source of carbon and energy, including the metabolites formed by M. luteus, the nature of the metabolites formed by *P. oleovorans* remained unknown (27). We therefore started with a comprehensive characterization of B[*a*]P metabolites formed by the latter using carbon-limited batch cultures.

### *P. oleovorans* B[*a*]P metabolites.

The analytical examination of the respective culture supernatants confirmed this strain’s ability to partially metabolize B[*a*]P (26). The dominant metabolites formed were B[*a*]P-1,6-dione, B[*a*]P-7,8-dione, B[*a*]P-7,10-dione, and 3-OH-B[*a*]P (Fig. S4A). Altogether, a total of 8 metabolites could be identified, 3 of which seem to be specific for this particular organism (Fig. S4B and C). Among them were 8-OH-B[*a*]P, B[*a*]P-7,10-dione, and an additional monohydroxylated B[*a*]P, which could not be further specified (Fig. S4D).

10.1128/mBio.01223-21.5 FIG S4 The B[*a*]P metabolites of *P. oleovorans*. Presented are the metabolites determined by GC-MS with the respective retention time (rt/min), together with quantifier and qualifier ions and the corresponding concentrations (A). GC-MS chromatograms and corresponding full-scan mass spectra of control (medium with 100 μM B[*a*]P) (B) and metabolites formed by *P. oleovorans* on day 5 of cultivation (C). Mass spectra of the unknown X-OH-B[*a*]P (20.5 min) and (D) the corresponding mass spectrum of the standard compound 3-OH-B[*a*]P (20.2 min). Download FIG S4, JPG file, 1.1 MB. Copyright © 2021 Lemoine et al. 2021 Lemoine et al. https://creativecommons.org/licenses/by/4.0/ This content is distributed under the terms of the Creative Commons Attribution 4.0 International license.

### Analytical characterization of commensal B[*a*]P metabolism *in situ* and effect on BPDE-DNA adduct formation.

With the PAH metabolite patterns of both microorganisms established, it was possible to follow B[*a*]P metabolism and distribution of resulting metabolites in the colonized skin model. For this purpose, B[*a*]P was topically applied to stably established microbial competent 3D skin cultures for 7 days. Following substance treatment, the concentration of B[*a*]P and its metabolites was measured starting from the surface throughout the model on day 8, with analyte levels in the supporting culture medium being monitored over the entire cultivation period (Data Set S1).

**FIG 1:**
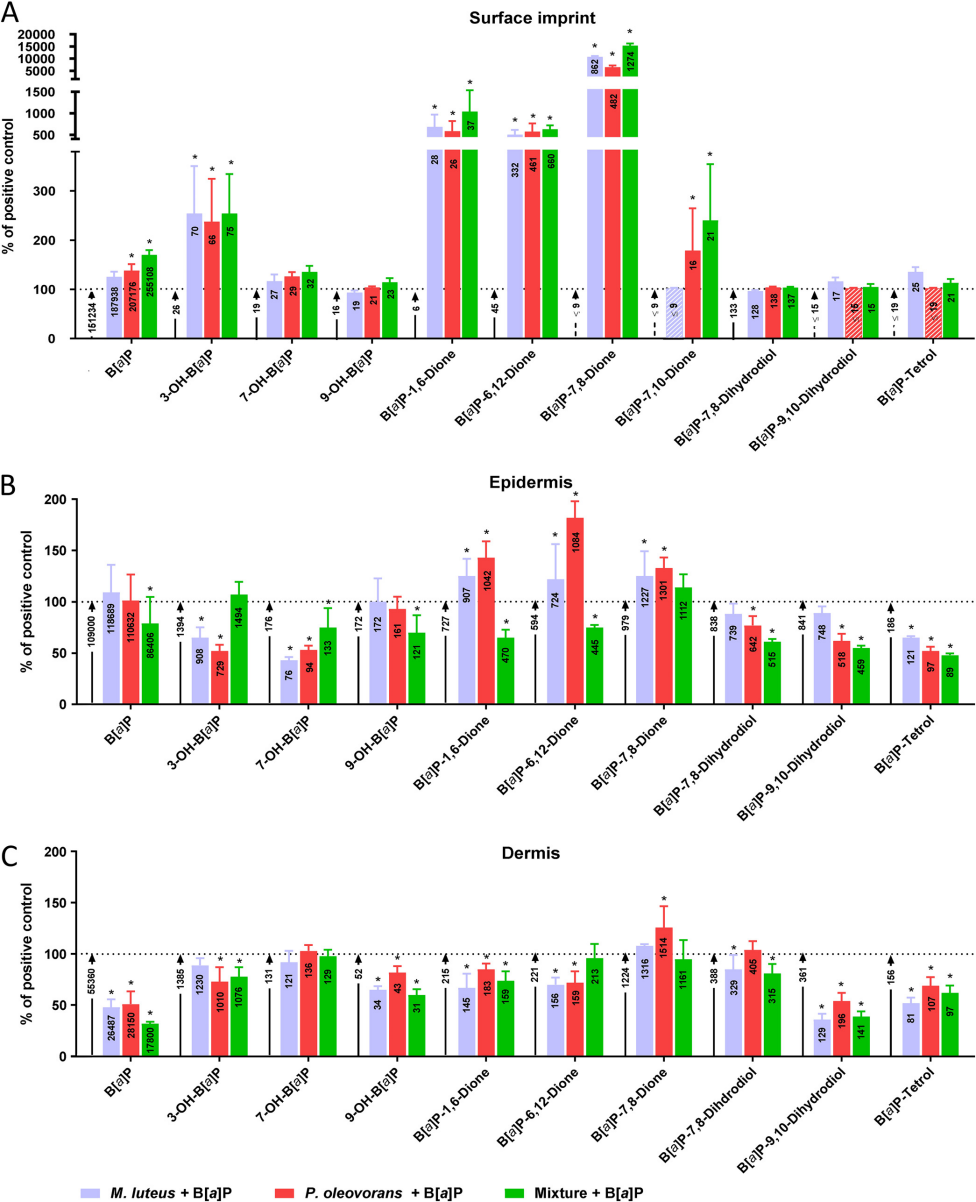
Concentrations of B[*a*]P and its metabolites from the surface imprint (A), epidermis (B), and dermis (C) of microbial skin tissue coculture colonized as indicated after 7 days of repeated B[*a*]P treatment, as determined via GC-MS. Each bar represents 6 biological replicates (mean + SD) relative to the B[*a*]P-treated uncolonized control. Each bar depicts the measured substance concentrations in pmol/skin model. The concentration of the B[*a*]P-treated control lacking a microbiota is indicated in the arrow next to the respective bar. For substances that could not be measured, the concentration is indicated as being below the smallest measured concentration in calibration, and corresponding columns or arrows are hatched. The observed differences are considered significant (*, *P* < 0.05).

The data indicate that commensal B[*a*]P metabolism occurs directly at the surface with the cocolonized models, which also retained larger amounts (2 of 3 were significantly higher) of unmetabolized B[*a*]P than uncolonized 3D skin (Fig. S5A). Furthermore, the concentrations of all detected B[*a*]P-diones as well as 3-OH-B[*a*]P were strongly elevated, all of which were established metabolites of at least one of the two commensals. B[*a*]P-7,10-dione was formed only in the presence of *P. oleovorans* (Fig. S5B). Notably, concentrations of the eukaryotic B[*a*]P-7,8,9,10-tetrahydrotetrol (B[*a*]P-tetrol; i.e., the hydrolysis product resulting from *trans*-opening of the epoxide moiety in BPDE) were slightly elevated in the presence of M. luteus (Fig. 1A).

10.1128/mBio.01223-21.6 FIG S5 GC-MS chromatograms of the surface imprint of cocultures colonized as indicated and the control (A). GC-MS ion chromatograms with an *m/z* of 428, indicating metabolically formed B[*a*]P-7,10-dione from *P. oleovorans* (B). Download FIG S5, JPG file, 0.3 MB. Copyright © 2021 Lemoine et al. 2021 Lemoine et al. https://creativecommons.org/licenses/by/4.0/ This content is distributed under the terms of the Creative Commons Attribution 4.0 International license.

Except for B[*a*]P-7,10-dione, all of the above-mentioned metabolites were also detectable in the epidermis and dermis. The predominant species were B[*a*]P-1,6-dione, B[*a*]P-6,12-dione, and B[*a*]P-7,8-dione or just B[*a*]P-7,8-dione, respectively (Fig. 1B and C). For all monitored metabolites, there was a clear colonization-dependent impact, indicating a distinct microbiome-mediated effect (Fig. S6A to C). In addition, the epidermis of models cocolonized with both organisms tended to contain lower concentrations of B[*a*]P-diones than the surface. In the deeper dermal layers, this effect was less pronounced.

10.1128/mBio.01223-21.7 FIG S6 GC-MS chromatograms of the colonized skin models and the basal cell culture medium. Chromatogram of the uncolonized skin treated with B[*a*]P (A), the M. luteus coculture (B), and the *P. oleovorans* coculture (C) on day 8 of cultivation of the epidermis and the dermis. GC-MS ion chromatograms with *m/z* of 404, indicating metabolically formed B[*a*]P-tetrol. Mass spectra of B[*a*]P-tetrol (36.7 min) injected as an analytical standard and extracted from the cocultures from the dermis (E). GC-MS chromatograms of the basal cell culture medium (F) spiked with standard substances (calibration mixture), of the uncolonized skin treated with B[*a*]P, the M. luteus coculture, the *P. oleovorans* coculture, and the mixed coculture on day 8 of cultivation. Shown is a section of the superimposed chromatogram from 26.50 to 42.50 min. The internal standards (IS) are numbered according to Table 1. Download FIG S6, JPG file, 1.0 MB. Copyright © 2021 Lemoine et al. 2021 Lemoine et al. https://creativecommons.org/licenses/by/4.0/ This content is distributed under the terms of the Creative Commons Attribution 4.0 International license.

Notably, commensal colonization led to significantly reduced levels of B[*a*]P-9,10-diol and B[*a*]P-tetrol in all investigated epidermal and dermal samples (Fig. S6D and E). Also, the concentration of unmetabolized B[*a*]P was greatly reduced in the dermis of commensal models, with the strongest effect occurring in the mixed coculture (Fig. 1B and C; Fig. S6C).

Several of the metabolites passed through the entire model and were detectable in the basal cell culture medium. These were B[*a*]P-tetrol, B[*a*]P-9,10-diol, B[*a*]P-7,8-diol, 3-OH-B[*a*]P, B[*a*]P-1,6-dione, and B[*a*]P-6,12-dione, which initially appeared 24 h after the first application of B[*a*]P to the model surface, and 7-OH-B[*a*]P, which appeared after 48 h (Table S1A). The corresponding concentrations increased in a time-dependent manner until reaching a plateau phase from day 5 onward, with the samples of the colonized models featuring significantly lower levels of B[*a*]P and B[*a*]P metabolites (Fig. S6F). This effect was particularly pronounced with M. luteus.

10.1128/mBio.01223-21.10 TABLE S1 B[*a*]P and its metabolites in microbial skin tissue coculture. (A) Sum of the concentrations of B[*a*]P and its metabolites (nanomolar) excreted into the cell culture medium over the entire cultivation period of 8 days. (B) Amount of B[*a*]P and its metabolites as well as their sums in control tissue, M. luteus coculture, *P. oleovorans* coculture, and mixed coculture, each treated with B[*a*]P, as a sum of all compartments (skin surface, epidermis, dermis, and cell culture medium) determined by GC-MS, in picomoles per skin model. In addition, the corresponding sums of the metabolite amount are given in parentheses as a percentage of the amount of B[*a*]P applied throughout the cultivation period (350 nmol/skin model). Overall, the recovery rate of B[*a*]P and its metabolites relative to the amount of B[*a*]P applied ranged from 95 to 107%. If a substance could not be detected, it is indicated as being below the smallest measured concentration in the calibration (bsc). Download Table S1, JPG file, 0.8 MB. Copyright © 2021 Lemoine et al. 2021 Lemoine et al. https://creativecommons.org/licenses/by/4.0/ This content is distributed under the terms of the Creative Commons Attribution 4.0 International license.

**FIG 2:**
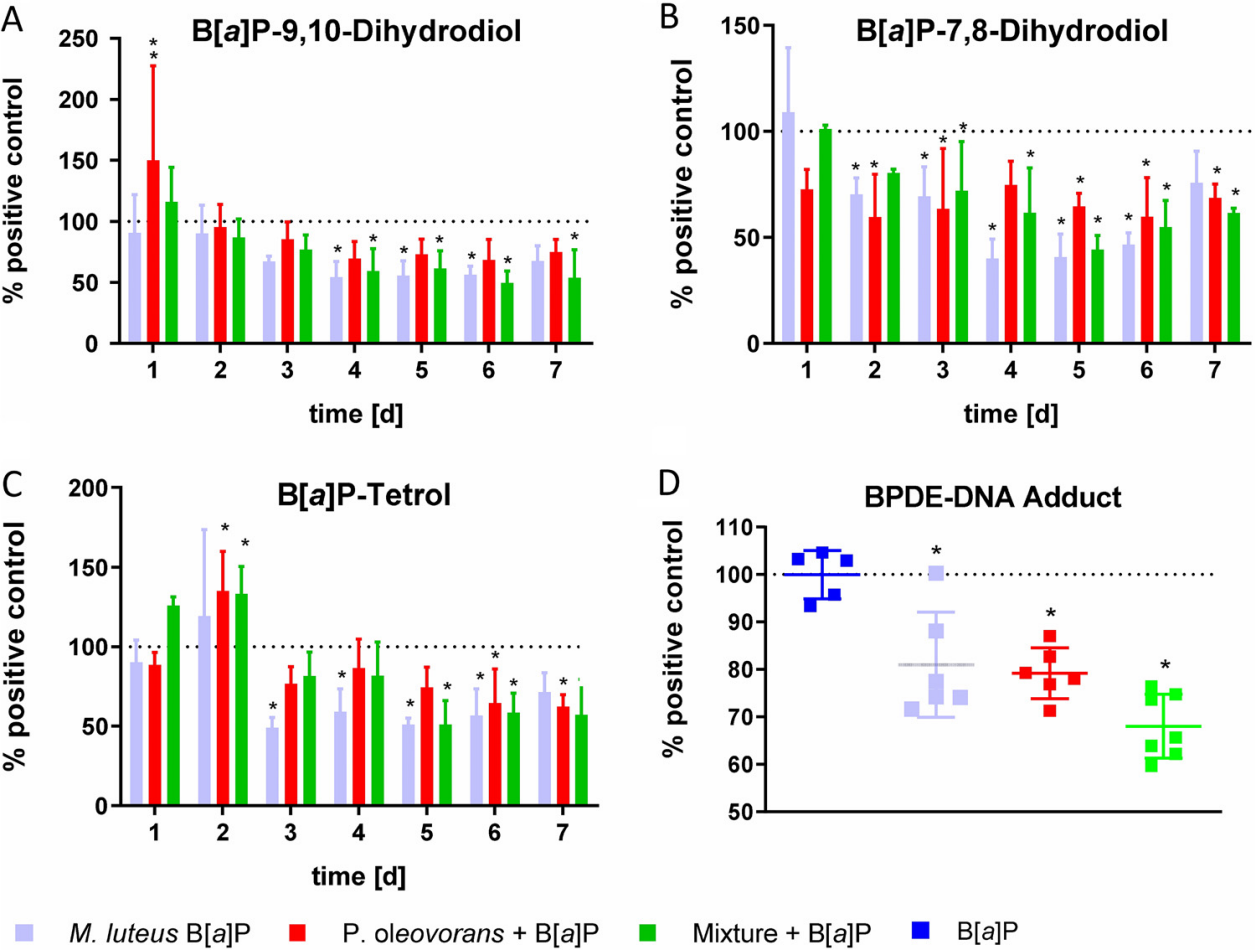
Concentrations of 3 representative B[*a*]P metabolites in the basal culture medium of microbial skin tissue cocultures over 7 days of repeated B[*a*]P treatment, as determined via GC-MS. Each bar represents 6 biological replicates. (A) B[*a*]P-7,8-dihydrodiol, (B) B[*a*]P-9,10-dihydrodiol, and (C) B[*a*]P-tetrol levels (mean + SD) as percentages of the B[*a*]P-treated uncolonized control. (D) Number of BPDE-DNA adducts of the microbial skin tissue cocultures colonized as indicated, compared to the B[*a*]P-treated control lacking a microbiota. Epidermis and dermis were examined as a whole. The observed differences are considered significant (*, *P* < 0.05).

In concordance with this and with what was found for B[*a*]P-9,10-diol and B[*a*]P-tetrol (that is, the significant reduction of metabolite levels in the microbe-colonized models), BPDE-DNA adducts were also found to be strongly reduced in models subjected to commensal colonization (Fig. 2). Meanwhile, controls without B[*a*]P showed no BPDE-DNA formation, irrespective of their microbial competence.

### Commensal impact on skin function.

**FIG 3:**
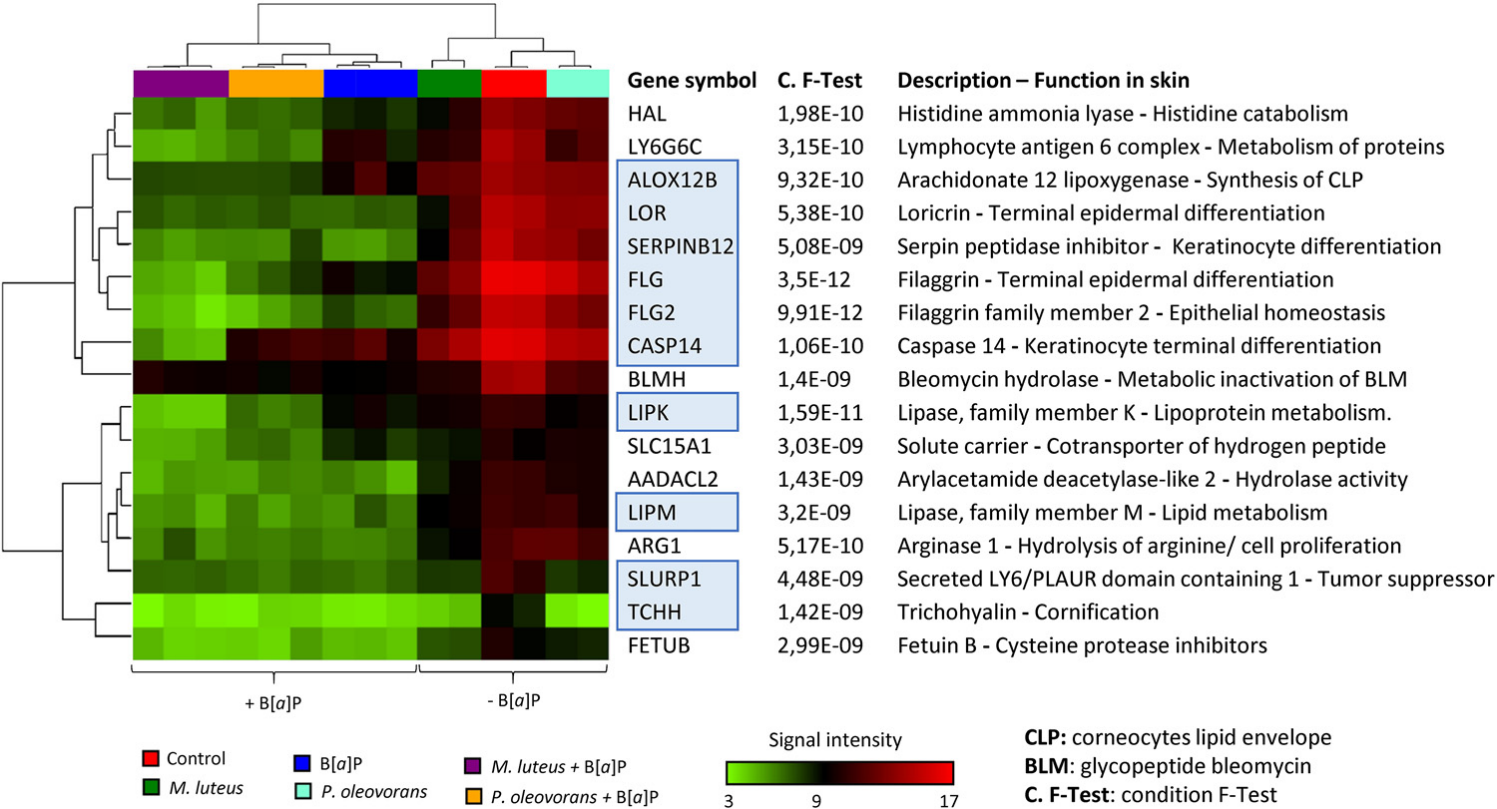
Hierarchical clustering of the 17 most significantly regulated genes according to the conditions of the F test in microbial skin tissue coculture with and without B[*a*]P treatment. Shown are the signal intensities of the transcripts measured using Clariom S microarray analysis. Ten of these 17 genes code for proteins that are related to epidermal differentiation and are marked in blue. In addition, a description and the most likely function in the skin are presented for each gene listed.

The potential functional impact of commensal B[*a*]P-metabolism on the host and skin function was studied using transcriptomic assays. The Clariom S system records the activity of more than 20,000 well-annotated transcripts, hence providing coverage of most of the functionally annotated genes. Compared to uncolonized controls, treatment of colonized models with B[*a*]P significantly affected more than 2,500 transcripts, while substance treatment alone had an effect on 1,300 transcripts (Fig. S7). Comparison among all treatment groups showed that 10 of the 17 most influenced transcripts strikingly encode proteins that play a role in epidermal differentiation, all of which were repressed in cocolonized models as well as after treatment with B[*a*]P (Fig. 3). This includes several epidermal differentiation complex (EDC) genes, such as those encoding filaggrin (FLG), loricrin, and caspase 14.

10.1128/mBio.01223-21.8 FIG S7 Number of differentially expressed genes in skin colonized with M. luteus, *P. oleovorans*, and their mixture after B[*a*]P application compared to B[*a*]P treatment alone. Additionally, the transcriptional response of EpiDermFT models on day 8 of microbial colonization with and without B[*a*]P application. Mapping 76.3% of the available transcripts PCA shows separation of all terms of cocultivation and controls with and without B[*a*]P treatment. Download FIG S7, JPG file, 1.1 MB. Copyright © 2021 Lemoine et al. 2021 Lemoine et al. https://creativecommons.org/licenses/by/4.0/ This content is distributed under the terms of the Creative Commons Attribution 4.0 International license.

**FIG 4:**
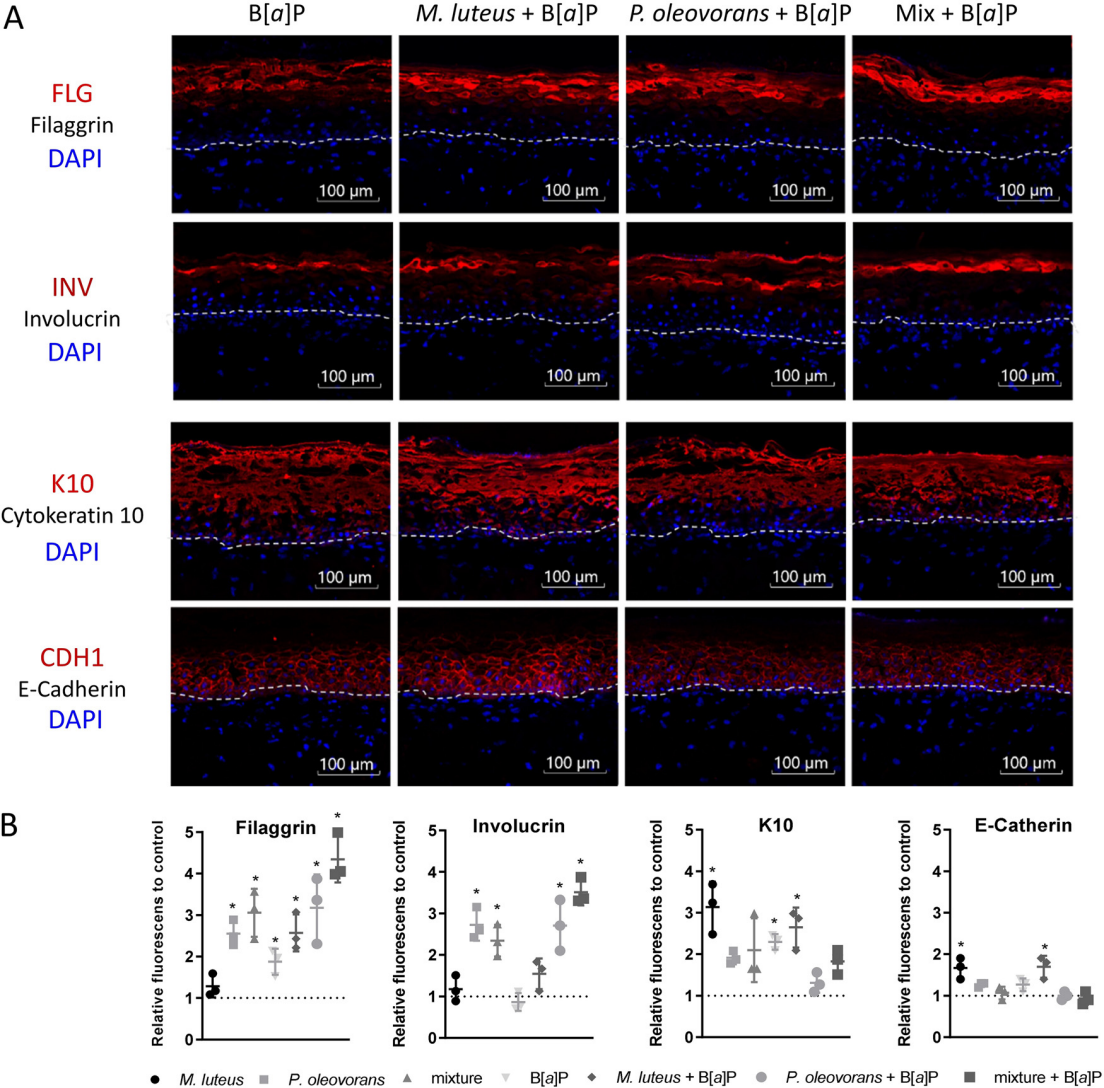
Expression of epidermal differentiation markers in microbial skin tissue cocultures with and without B[*a*]P treatment. (A) Immunostaining of selected differentiation markers and a tight junction marker of skin sections of microbial competent skin models, colonized as indicated and with B[*a*]P treatment. All antibody stains are red, and the cell nuclei stained blue with DAPI. The dashed line indicates the course of the basal lamina. (B) Summary of the immunostaining of 3 biological replicates relative to the uncolonized skin, including all controls without B[*a*]P application. The observed differences are considered significant (*, *P* < 0.05).

The results of the transcriptome analysis strongly imply a potential commensal influence on skin differentiation, particularly following substance treatment. Subsequent analysis therefore focused on the fluorescent visualization of various functional markers for different stages of differentiation as well as a tight junction (TJ) marker (Fig. 4A and Fig. S8). In the B[*a*]P-exposed models, bacterial colonization led to a strong increase in the late differentiation markers FLG and involucrin (IVL), with a particularly strong increase for *P. oleovorans* and mixed skin tissue (Fig. 4A and B).

10.1128/mBio.01223-21.9 FIG S8 Expression of epidermal differentiation markers in microbial skin tissue cocultures without B[*a*]P treatment. Exemplary shown is an immunostaining of selected differentiation markers and a tight junction marker of skin section of microbial competent skin models, colonized as indicated without B[*a*]P treatment. Download FIG S8, JPG file, 0.8 MB. Copyright © 2021 Lemoine et al. 2021 Lemoine et al. https://creativecommons.org/licenses/by/4.0/ This content is distributed under the terms of the Creative Commons Attribution 4.0 International license.

**FIG 5:**
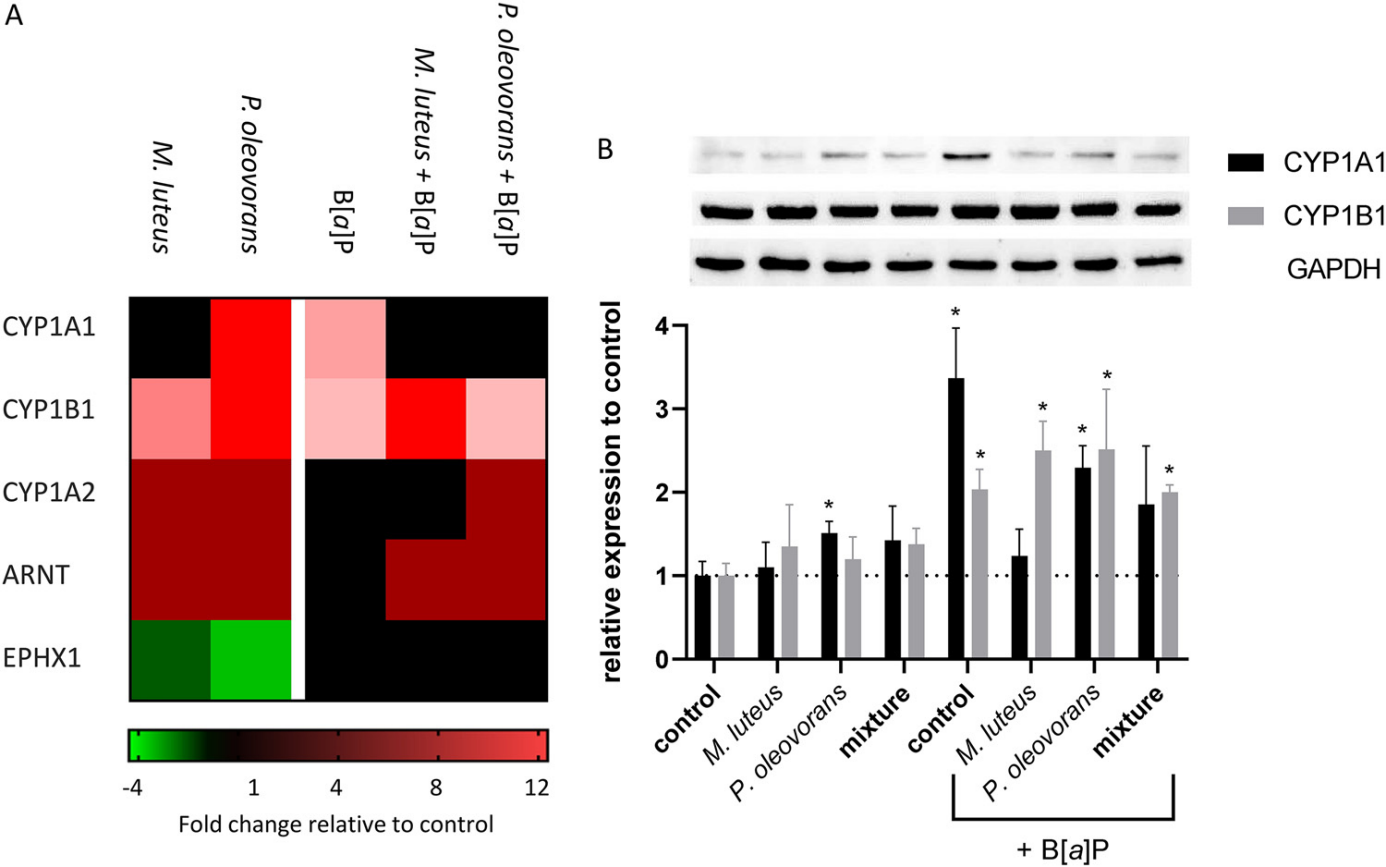
Expression of enzymes involved in B[*a*]P activation in microbial skin tissue coculture. (A) Cluster map showing the fold change compared to uncolonized skin at day 8 of microbial colonization. Shown are the gene symbols of significantly differentially expressed genes with a *P* value of ≤0.05. (B) Immunoblot of total protein of microbial skin tissue coculture, colonized as indicated, as well as the summary of the immunoblots of 3 biological replicates relative to the uncolonized skin. The observed differences are considered significant (*, *P* < 0.05).

In light of the skin’s dual function as a physicochemical barrier, we further investigated the commensal impact on the expression of several key proteins involved in human B[*a*]P metabolism. Enzymatic expression of CYP1A1 was found to be decreased in the commensal models following treatment with B[*a*]P, while there was no clear effect on CYPs 1B1 and 1A2 or epoxide hydrolase 1. In contrast, both microorganisms led to increased expression of the regulatory cofactor aryl hydrocarbon receptor nuclear translocator (ARNT), even in the absence of B[*a*]P (Fig. 5A and B).

**FIG 6:**
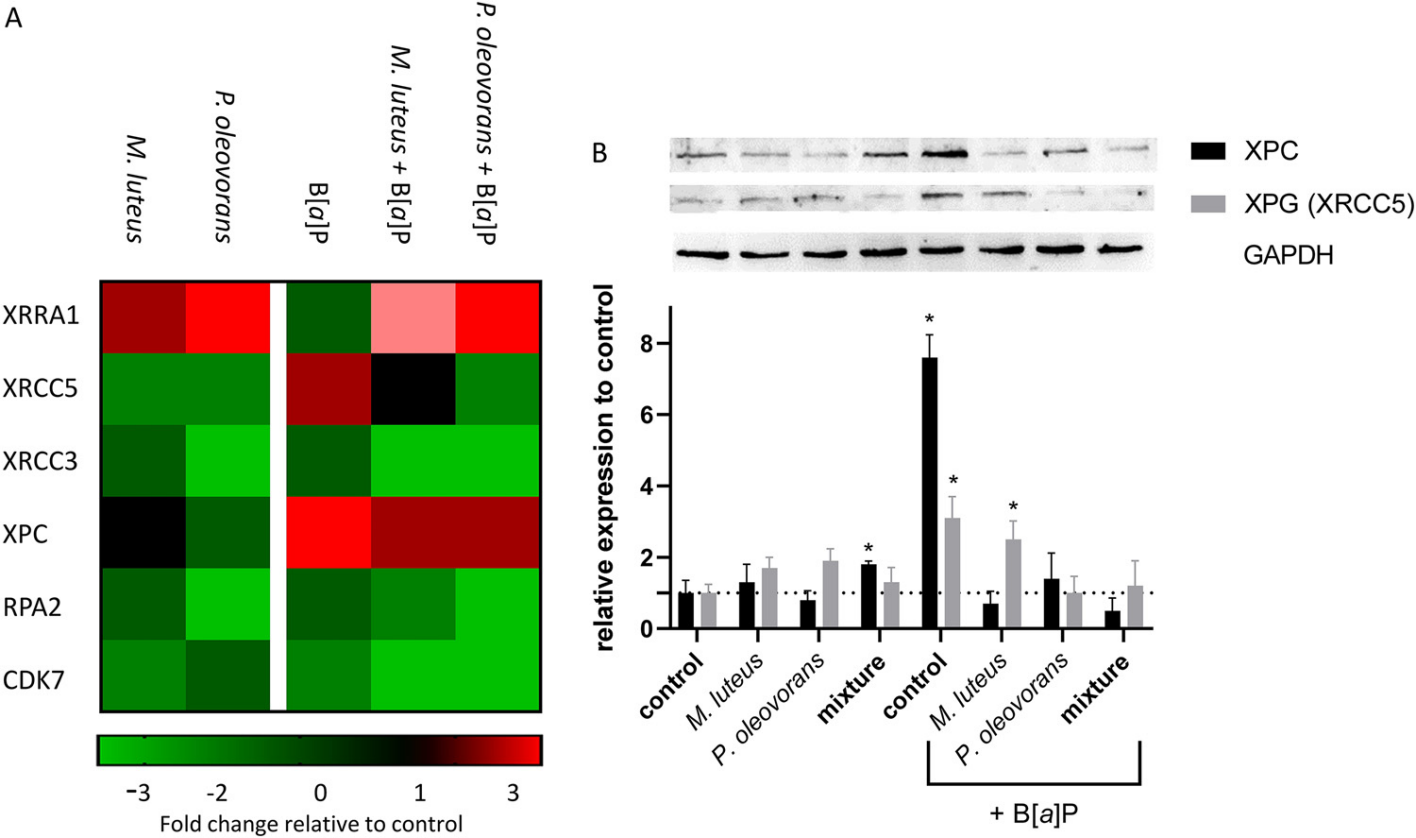
Expression of enzymes of the nucleotide excision repair (NER) pathway in microbial skin tissue coculture. (A) Cluster map showing the fold change compared to uncolonized skin at day 8 of microbial colonization. Shown are the gene symbols of significantly differentially expressed genes with a *P* value of <0.05. (B) Immunoblot of total protein of microbial skin tissue coculture, colonized as indicated, as well as the summary of the immunoblots of 3 biological replicates relative to the uncolonized skin control. The observed differences are considered significant (*, *P* < 0.05).

Metabolic activation of B[*a*]P in eukaryotes can lead to the formation of bulky DNA adducts that can be repaired by the nucleotide excision repair pathway (NER). The treatment of 3D skin models with B[*a*]P resulted in an increase in the expression of xeroderma pigmentosum complementation group C (XPC) and XPG proteins, both of which are typical representatives of this repair pathway (Fig. 6B). Following B[*a*]P treatment, however, their gene and protein expression was significantly reduced in microbe-colonized skin tissue compared to the control treated with B[*a*]P only (Fig. 6A and B).

## DISCUSSION

Growing evidence suggests potential chemical-associated effects of microbiome-host interactions on human health (7, 32, 33). However, toxicity studies are still largely conducted without particular consideration of the microbiota (34). At best, the microbiota is considered a systemic compartment when toxicity is tested *in vivo*. However, this does not consider the high species specificity of microbiota, nor does it acknowledge that even without this species specificity the microbiotas of laboratory animals are strongly impacted by the artificial environments the animals are housed in and thus are hardly representative (31). In the context of risk assessment of xenobiotic exposure, this not only is scientifically unsatisfying but also carries a true risk of missing relevant health effects. However, apart from a partial lack of awareness of this issue, another major point has been a lack of appropriate models to systematically study such effects. This is true for the gut but also for the skin (7, 35). For dermal environments the recent development of our microbially competent coculture model partly overcomes this, as it makes it possible to study microbially driven modulation of B[*a*]P metabolism and toxicity and its effects on the host (31). The results confirm that the previously observed commensal metabolism of B[*a*]P also occurs *in situ* (26, 27). However, and more importantly, they provide a first glance at the corresponding metabolite patterns along skin layers and how this potentially affects host toxicity in a complex and hitherto unexpected manner.

Skin colonization leads to an accumulation of unmetabolized B[*a*]P and bacterial metabolites, including B[*a*]P-1,6-dione, B[*a*]P-6,12-dione, B[*a*]P-7,8-dione, B[*a*]P-7,10-dione, and 3-OH-B[*a*]P (27), on the skin surface and, partially, in the epidermis. The strong increase of these commensal metabolites points to microbiotic recruitment of B[*a*]P as a source of carbon and energy and is in line with previous *in vitro* studies (26, 36). This is particularly evident in the case of B[*a*]P-7,10-dione, which, being a metabolite of *P. oleovorans*, occurred exclusively on the surface of the correspondingly colonized skin models. However, the identification of B[*a*]P-tetrol and B[*a*]P-9,10-dihydrodiol on the surface of skin harboring M. luteus shows that concomitant human phase I metabolism occurs, as neither of the two metabolites occurred in bacterial batch cultures (27). Notably, when assessed throughout their various compartments, the colonized models showed a slight overall decrease of B[*a*]P metabolites compared to uncolonized skin (∼4.5%), which was significant for a majority of the metabolites. This included B[*a*]P-tetrol, where the respective strong reduction consequently points to fewer BPDE-DNA adducts being formed beforehand, an effect most pronounced in the presence of *P. oleovorans* and its mixed coculture. Likewise, the expression of key enzymes of the NER (XPC and XPG) was reduced in microbial skin tissue cocultures. In addition, the amount of B[*a*]P-tetrol as well as other metabolites passing through the model into the basal medium was significantly reduced in the commensal models, with M. luteus leading to nearly 50% reduction. This indicative beneficiary commensal effect is in contrast to what was observed previously with batch cultures, where bacterial metabolism of B[*a*]P actually enhanced B[*a*]P-mediated genotoxicity (27). Given that the colonized models show production of the same metabolites *in situ*, the effect observed here came as a surprise.

Together, the results therefore strongly point to commensally induced changes in skin metabolism and/or barrier function. Overall, the analytical results of the uncolonized skin models match those of a previous study by Brinkmann et al. (37), who showed that MatTek’s EpiDermFT metabolizes B[*a*]P to form typical human metabolites. However, in the uncolonized models, only a small fraction of B[*a*]P is metabolized due to the low or absent metabolic activity of corneocytes. Only about 5.2% of the applied B[*a*]P was metabolized, with the remaining B[*a*]P accumulating on the skin surface and in the epidermis (Table S1B). Since lipophilic compounds steadily penetrate the skin by passive diffusion, this induces the buildup of a substance reservoir (38). Befittingly, for the uncolonized models, the major amount of the B[*a*]P was indeed found to be metabolized in the metabolically active epidermis. In agreement with the work of Bourgart et al. (39), the more hydrophilic metabolites, such as B[*a*]P-tetrol, B[*a*]P-7,8-diol, and B[*a*]P-9,10-diol, subsequently penetrated throughout the skin, while B[*a*]P predominantly remained in the upper layers (39). Meanwhile, the colonized models are also metabolically active but feature increased accumulation of B[*a*]P and known bacterial metabolites on the surface as well as showing an altered layer-specific decrease. On the transcriptomic level, this was accompanied by strong changes in key transcripts involved in epidermal differentiation.

The general observation of commensals influencing skin differentiation matches previous results from other studies, including animals (40). The most strongly altered transcripts include LIPM and LIPK, both of which have an essential function in lipid metabolism of the most differentiated epidermal layers (41). Another heavily affected transcript is CASP14, which is a predominant caspase in the epidermal SC and is required for cornification (42). The reduced transcript levels are in line with the age of the models at the time of sampling. Instead, selected corresponding protein markers of skin differentiation were revealed to be elevated. Such an apparent commensal-related strengthening of the skin barrier has indeed been observed before, although not in a toxicological context and without such a strong modulatory effect on systemic substance metabolism and availability (43, 44). Our results show that *P. oleovorans* had a particularly strong influence, promoting terminal differentiation as exemplified by an increase in the expression of FLG and IVL. In contrast, colonization with M. luteus leads to less pronounced changes, showing a slight but significant enhancing effect on FLG, K10, and the TJ marker E-cadherin. Simultaneous colonization of the 3D skin models with both organisms revealed the greatest increases in expression of IVL and FLG, supporting the finding of Loomis et al. (44) that FLG expression is more strongly enhanced by multiple commensals than by a single species. The large amount of unmetabolized B[*a*]P on the surface of the mixed microbial skin tissue cocultures suggests that FLG and IVL particularly contribute to a reduction in the diffusion of chemicals into the skin. This is in agreement with the results of Joensen et al. (45), who showed that loss-of-function mutations of FLG lead to increased internal exposure to phthalate metabolites. IVL is also important for skin permeability to chemicals, with a triple knockout of IVL, envoplakin, and periplakin resulting in a defective epidermal barrier (46). Moreover, tissue permeability is regulated by TJs and keratin filaments (47), whose expression can be enhanced by the presence of commensal and probiotic strains (48). In our models, M. luteus particularly induced E-cadherin and K10. It seems reasonable to assume that the resulting strengthening of the epidermis is causative of the respective strong effects on metabolite concentrations in the dermis and basal cell culture medium, respectively. At the same time, commensally induced increased terminal differentiation of the SC (i.e., as seen for *P. oleovorans*) promotes the accumulation of B[*a*]P on the skin surface, thus reducing the rate of epidermal skin-type B[*a*]P metabolism. As this strongly affects levels of B[*a*]P-9,10-diol, B[*a*]P -7,8-diol, and BPDE, it would obviously reduce the number of BPDE-DNA adducts in general.

The altered barrier function also systemically affected key genes of human phase I metabolism. Effects were seen particularly for the AhR signaling pathway, namely, ARNT, CYP1B1, and CYP1A1, with the effect being particularly strong on the latter. Although CYP1A1 expression is increased in coculture with *P. oleovorans*, it is significantly reduced after repeated B[*a*]P application in all studied microbial cocultures compared to uncolonized skin. This is likely attributed to the smaller amount of B[*a*]P penetrating the SC, which in turn reduces the available concentration in the metabolically active part of the epidermis. In the context of B[*a*]P toxification, the commensal influence on the skin barrier hence apparently outweighs any microbially induced changes in CYP expression. Our results clearly show that commensals are able to metabolize as well as potentially toxify B[*a*]P on and in human skin. However, with regard to B[*a*]P genotoxicity, the microbial effects on host skin differentiation have a strong modulating beneficial influence due to an enhanced physicochemical barrier function. The latter alterations cause a commensal-related reduction of the toxicity of B[*a*]P especially by inhibiting the uptake of this procarcinogen and its subsequent metabolic activation. The microbial influence on epithelial permeability and integrity has important implications for the absorption, metabolism, and distribution of xenobiotics and environmental pollutants such as B[*a*]P. Overall the results demonstrate the respective microbe-host interactions to have much more complex effects on substance toxification and metabolism than previously inferred from studies on single systems. Depending on the particular substance and circumstances, this could lead to either over- or underestimation of toxicity, both of which should be avoided. Therefore, there is an urgent need to address the effect of the microbiome on potential systemic host toxicity more systematically. Given the species specificity of the microbiome, this has to be implemented using commensally competent testing systems that are specific for the human host.

## MATERIALS AND METHODS

Skin models obtained from MatTek rely on ethically sourced materials from accredited institutes and are subject to strict donor consent. Their suppliers have represented in writing that they are strictly regulated by and comply with U.S. federal government standards.

### Chemicals and media.

Gas chromatography-mass spectrometry (GC-MS)-grade solvents were purchased from Thermo Fisher Scientific (Waltham, MA, USA). All other chemicals were sourced from Sigma-Aldrich (St. Louis, MO, USA) unless otherwise noted. EpiDermFT tissue models with lot numbers 29346, 25358, 25392, 29308, and 29388 and associated maintenance medium were from MatTek (Ashland, MA, USA). Analytical standards were purchased from MRIGlobal Chemical Carcinogen Repository (Kansas City, MO, USA): B[*a*]P, 7-OH-B[*a*]P, 9-OH-B[*a*]P, B[*a*]P-*trans*-7,8-dihydrodiol(±), B[*a*]P-*trans*-9,10-dihydrodiol(±), B[*a*]P-1,6-dione, B[*a*]P-6,12-dione, B[*a*]P-7,8-dione, B[*a*]P-*r*-7,*t*-8,*t*-9,*c*-10-tetrahydrotetrol, benzo[*a*]anthracene-*cis*-5,6-dihydrodiol (B[*a*]A-*cis*-5,6-dihydrodiol), 5-methylchrysene-*r*-1,*t*-2,3,*c*-4-tetrahydrotetrol, and deuterated B[*a*]P-D_12_. 3-OH-B[*a*]P, 3-OH-B[*a*]P-^13^C_6_, and B[*a*]P-1,6-dione were from Toronto Research Chemicals (North York, ON, Canada).

### Bacterial isolates and bacterial growth.

Cocultures were set up as previously described, with 10^4^ to 10^6^ cells of Micrococcus luteus 1B and/or Pseudomonas oleovorans 1C used for skin inoculation (31).

Beforehand, to determine the B[*a*]P metabolites of *P. oleovorans*, the latter was cultured in minimal medium (MM) supplemented with 100 μM B[*a*]P as previously described for M. luteus by Sowada et al. (27). Cell harvesting was performed daily over a period of 2 weeks. For later analytical examination, 25 ml of the bacterial culture supernatant was frozen and stored at −20°C.

### Tissue viability assay for selection of the applied B[*a*]P concentration.

As the basis for our coculture, we used the full-thickness skin model EpiDermFT from MatTek (Ashland, MA, USA). Directly after delivery, the models were transferred into six-well plates (Greiner Bio-One, Frickenhausen, Germany) with 2.5 ml of antibiotic-free EPI-100-MM-ABF maintenance medium and cultivated for 24 h to recover at 37°C and 5% CO_2_ as recommended by the manufacturer. Before treatment of the actual microbial skin tissue coculture with B[*a*]P, we first evaluated 3 different concentrations of B[*a*]P (5 nM/cm^2^, 50 nM/cm^2^, and 500 nM/cm^2^) and determined their influence on tissue viability via an MTT [3-(4,5-dimethyl-2-thiazolyl)-2,5-diphenyl-2H-tetrazolium bromide] assay. A high B[*a*]P concentration can promote bacterial use as a food source but could impair the viability of the skin cells and thus inhibit its B[*a*]P metabolism. The MTT assay was performed using MatTek’s MTT concentrate, MTT diluent, and MTT extractant according to the manufacturer’s instructions. The skin models (1-cm^2^ diameter) were treated daily with one of the 3 B[*a*]P concentrations prepared in acetone of analytical grade or solvent control (pure acetone), respectively. Acetone was chosen as the deposition vehicle due to its successful use in other skin B[*a*]P metabolism studies (37, 39, 49). The cell culture medium was exchanged daily, and the MTT assay was carried out after 3 and 7 days of B[*a*]P treatment. With moderate viability losses of 15% after 7 days of B[*a*]P application, the mean concentration of 50 nM/cm^2^ was chosen for further experiments (Fig. S1).

10.1128/mBio.01223-21.2 FIG S1 Glycolytic activity of MatTek’s EpiDermFT models after 4 and 7 days of B[*a*]P application (3D skin was treated with 3 different B[*a*]P concentrations, as indicated). Download FIG S1, JPG file, 0.5 MB. Copyright © 2021 Lemoine et al. 2021 Lemoine et al. https://creativecommons.org/licenses/by/4.0/ This content is distributed under the terms of the Creative Commons Attribution 4.0 International license.

### Tissue culture and bacterial quantification.

The EpiDermFT models were routinely cultivated and subjected to bacterial inoculation or solvent treatment as described earlier (31). Bacterial inoculation of the respective models was performed using three droplets of bacterial suspension at a volume of 5 μl each, with the droplets evenly spaced across the model surface and then left to dry. At 24 h after establishing the coculture of the B[*a*]P-degrading skin commensals, 50 nM/cm^2^ B[*a*]P was applied as evenly as possible on the surface of the skin tissues (diameter, 1 cm^2^). Identical amounts of solvent were applied in control experiments. This procedure was repeated daily at the same time during the cultivation period of 8 days, stopping 24 h before harvesting the EpiDermFT models. In total, each model was treated with B[*a*]P for 7 consecutive days, resulting in a total concentration of 350 nmol/skin model. Tissues and the daily collected basal cell culture medium were separately stored at −80°C until further processing.

Bacterial growth in coculture was quantified using plate counts, since there was no significant added value in using quantitative PCR (qPCR) additionally (31). As in our previous experiments, viable colony counts (CFU/cm^2^) showed stable quantities for M. luteus from day 4 of cultivation (Fig. S2A and C), while those of *P. oleovorans* increased slightly and were on average slightly higher. As reported previously, M. luteus appeared to grow exclusively on the SC, while *P. oleovorans* also penetrated into the dermis, although to a small extent (approximately ≤10%) (31). The cell numbers shown in Fig. 2 refer to those on the skin surface. In general, the B[*a*]P application had no significant influence on cell numbers (Fig. S2C). The selection of *P. oleovorans* and M. luteus as models for commensal B[*a*]P degraders followed both biological and practical considerations (31). Briefly, the selection covers species of both Gram variants as well as two of the major phyla of the skin’s microbiome. Both species are biologically relevant and have been repeatedly isolated from healthy subjects at different skin sites (26). Moreover, they are easily distinguishable from each other on agar plates and have a well-established potential for xenobiotic metabolism (26, 50).

10.1128/mBio.01223-21.3 FIG S2 Plate counts of skin models on days 0, 4, and 8 of bacterial colonization with and without B[*a*]P treatment. The individual points show the cell counts of one microbial skin tissue coculture each for M. luteus (A) and *P. oleovorans* (B). The horizontal bars show the medians of all measured values. The mean values of the plate counts of the mixed coculture (C) are shown in a table with a range of variation in percent. The respective plate counts on day 0 served as the starting cultures for all cocultures with and without subsequent B[*a*]P treatment. Download FIG S2, JPG file, 0.2 MB. Copyright © 2021 Lemoine et al. 2021 Lemoine et al. https://creativecommons.org/licenses/by/4.0/ This content is distributed under the terms of the Creative Commons Attribution 4.0 International license.

### Transcriptome analysis.

In order to gain insight into microbial-related changes in the skin’s gene expressions after B[*a*]P application, the skin tissues were examined as previously described by microarray analysis (31). For this purpose, total RNA was recovered subsequent to cell harvesting with a TissueLyser II (Qiagen, Hilden, Germany). The RNA was isolated with a TRIzol-based protocol using the TRIzol reagent (Thermo Scientific, Waltham, MA, USA) as described earlier (51). Microarray analysis was performed using duplicates of controls (uncolonized skin and M. luteus/*P. oleovorans* skin tissue coculture) and triplicates of treatments (B[*a*]P treatment and M. luteus/*P. oleovorans* with B[*a*]P application) in Clariom S human assays (Applied Biosystems, Foster City, CA, USA) at ATLAS Biolabs (Berlin, Germany). Data evaluation and interpretation were done using Transcriptome Analysis Console software v4.0.1.36 (TAC) (Applied Biosystems, Foster City, CA, USA) (*P* ≤ 0.05).

### Immunofluorescence.

To investigate changes in the epidermal barrier function, we snap-froze the skin tissues on day 8 of the cultivation in Tissue-Tek O.C.T. compound (Sakura Finetek, Torrance, CA, USA) using liquid nitrogen and stored these at −80°C until use. Tissues were cut into 7-μm-thick sections at −20°C using a Microm HM 550 cryostat and mounted on Super-Frost slides (both from Thermo Fisher Scientific, Waltham, MA, USA). Subsequently, cryosections were fixed for 10 min with ice-cold methanol (−20°C). Antibody (Ab) staining was performed as described by Hering et al. (52). The following antibodies were used: rabbit Ab against cytokeratin 10 (18343-1-AP) and involucrin (55328-1-AP; Proteintech Group, Rosemont, IL, USA) and mouse Ab against filaggrin (NBP2-53245-20; Novus Biologicals, Littleton, CO, USA) and E-cadherin (33-4000; Thermo Scientific, Waltham, MA, USA) at a concentration of 5 μg/ml. The appropriate secondary antibodies conjugated to Alexa Fluor 594 (red) (Molecular Probes, Eugene, OR) were applied (1:400 in phosphate-buffered saline with 0.1% Tween [PBST]) followed by Hoechst 33258 (Sigma-Aldrich, St. Louis, MO, USA) (1 μg/ml in Dulbecco's phosphate-buffered solution [DPBS]) application for counterstaining the nuclei. Sections were embedded in mounting medium and analyzed in biological triplicates using the LSM700 confocal microscope (Carl Zeiss, Oberkochen, Germany).

### Western blot analysis.

To determine potential changes in protein levels, Western blot analysis of selected proteins was carried out. The proteins were isolated in 100 μl of a urea buffer (8 M, pH 8.5) combined with lysis using a TissueLyser II (Qiagen, Hilden, Germany) operated for 5 min at 20 Hz. The cell residues were separated by centrifugation at a relative centrifugal force (rcf) of 10,000 for 10 min. Total protein concentration was evaluated using a bicinchoninic acid (BCA) assay (Thermo Fisher Scientific, Darmstadt, Germany). In general, 20 μg of total protein was then subjected to SDS-PAGE and transferred to nitrocellulose membranes following standard protocols. Primary antibodies against CYP1A1 (ABIN1872160; Antibodies-online Inc., Limerick, PA, USA), CYP1B1 (ABIN3184162), XPC (ABIN2855495), XPG (ABIN3187504), and glyceraldehyde 3-phosphate dehydrogenase (GAPDH) (ABIN2666338) were used for subsequent immunostaining, followed by visualization with appropriate horseradish peroxidase-coupled secondary antibodies (Santa Cruz Biotechnology) and enhanced chemiluminescence (34078; Thermo Scientific, Waltham, MA, USA) for detection in the Fusion FX6 Edge system (Vilber Lourmat, Eberhardzell, Germany).

### GC online-coupled to MS. (i) Analysis of *P. oleovorans* B[*a*]P metabolites.

To assess the bacterial influence on the human B[*a*]P metabolism, knowledge of the identity of the corresponding microbial B[*a*]P degradation products is crucial. For this reason, we analyzed the B[*a*]P metabolism of *P. oleovorans* via GC-MS strictly following a previously described protocol applied to M. luteus (27). This applies to the extraction, sample preparation, and GC-MS analysis. The verification was done using retention time (RT) and mass spectra (MS) of authentic references.

### (ii) B[*a*]P metabolism in microbial skin tissue coculture.

In order to determine the microbial influence on the B[*a*]P metabolite profile and distribution within the skin, the levels of B[*a*]P and its metabolites were quantified in surface imprint, tissue samples, and culture media from 6 biological replicates using GC-MS.

*(a) Cell lysis.* First, the surface imprint was taken as described earlier (31). The resulting cell pellet was digested by freeze-thawing. Three cycles of freezing cells in a dry ice-ethanol bath for 10 min and subsequent thawing at room temperature for 10 min were performed as described by de Bruin and Birnboim (53). For the subsequent metabolite extraction, the lysates were diluted with 750 μl PBS. The lysis of the skin tissues was done mechanically and enzymatically. Prior to cell lysis, the epidermis and dermis were separated, followed by homogenization with a TissueLyser II instrument twice for 5 min each at 20 Hz in 750 μl PBS each and digestion with 1 U/ml LiberaseTH enzymes (Roche, Mannheim, Germany) for 20 min at 37°C. The cell culture medium was used directly for metabolite extraction.

*(b) Metabolite extraction.* The surface imprint lysates, the skin tissue lysates, and the cell culture media (2.5 ml each) were spiked with a mixture of internal standards (Table 1) at a final concentration of 500 ng/ml (internal standard [IS] 2 = 1.8 μM, IS 3 = 1.9 μM, IS 4 = 1.9 μM, and IS 5 = 1.6 μM) in dimethyl sulfoxide (DMSO), except for B[*a*]P-D_12_, which was at 50 μg/ml (IS 1 = 181.1 μM) in DMSO (Table 1). The latter is due to the high B[*a*]P concentration in the samples, which is above the linear calibration range. In order to determine the B[*a*]P concentration, the samples were additionally measured in a 100-fold dilution in DMSO. Prior to extraction with dichloromethane (DCM) (1:7.5 volume ratio), ethanol (EtOH) was added as a disperser (54) (1:2.5 volume ratio). After the addition of EtOH and DCM, the samples were vortexed for 1 min. Subsequently, the samples were centrifuged for 15 min at 4°C and 4,000 × *g* for better phase separation. The lower organic phase containing the analytes was separated, the extraction was repeated twice, and the combined organic extracts were concentrated to dryness in 1.5-ml Eppendorf tubes using the Concentrator Plus (Eppendorf, Hamburg, Germany) at 30°C. The residues were dissolved in 20 μl *N*,*O*-bis(trimethylsilyl)-trifluoracetamide (BSTFA) (99% BSTFA with 1% trimethylsilyl chloride) (15222-10X1ML-F; Sigma-Aldrich, St. Louis, MO, USA) as a derivatization agent in GC vials (2-ml crimp top with a 300-μl inlet; Agilent Technologies, Santa Clara, CA, USA) to accomplish silylation of functionalized B[*a*]P metabolites (Fig. S3A) at 60°C for 90 min and shaking at 120 rpm in a water bath. Subsequently, 1 μl of each sample was diluted in 99 μl fresh BSTFA for quantification of unmetabolized B[*a*]P via GC-MS.

**TABLE 1:** Internal standards^*a*^

Substance	RT (min)	Quantifier *m/z*	Qualifier *m/z*	Designation
B[*a*]P-D_12_	26.6	264	262	1
3-OH-B[*a*]P-^13^C_6_	34.9	346	347	2
Chrysene 1,4-dione	29.0	404	405	3
B[*a*]A-*cis*-5,6-diol	22.7	406	316	4
5-Methylchrysene-1,2,3,4-tetrahydrotetrol	29.6	304	394	5

a Internal standards used for verification of B[*a*]P metabolite extraction and silylation. Designations correspond to the external standards listed in Table 2.

10.1128/mBio.01223-21.4 FIG S3 Structural formulas, molecular weights, and calibration curves of all investigated substance classes (OH-B[*a*]P, B[*a*]P-diol, B[*a*]P-dione and B[*a*]P-tetrol). Structural formulas and molecular weights of representative metabolites formed during derivatization with BSTFA (*N,O*-bis(trimethylsilyl)-trifluoracetamide) for GC-MS analysis. Shown are the respective silylated compounds (A). For B[*a*]P-tetrol, the dominant fragment formed upon cleavage during ionization is also shown (A). Calibration curves from epidermis treated with external standards are shown as examples (B). Each analyte was quantified in relation to the assigned internal standard (IS) (Table 1). For the epidermis and dermis, a four-point calibration was performed for cost reasons (matrix calibration). For the medium, an eight-point calibration was conducted. Download FIG S3, JPG file, 0.9 MB. Copyright © 2021 Lemoine et al. 2021 Lemoine et al. https://creativecommons.org/licenses/by/4.0/ This content is distributed under the terms of the Creative Commons Attribution 4.0 International license.

*(c) GC-MS measurements.* One microliter of silylated sample mixture was injected in splitless mode into a gas chromatograph 6890 (Agilent) equipped with an HP-5MS capillary column (30-m length, 250-μm inner diameter, 0.25-μm film thickness, 10-m precolumn; Agilent) coupled to a 5975 mass spectrometric detector (Agilent). Electron impact (EI) ionization was conducted at 70 eV. The data acquisition was performed in combined single-ion monitoring (SIM)/full scan mode in order to achieve high sensitivity for target analytes while at the same time acquiring information on unknown metabolites which might be included in the samples. The column was operated in constant-flow mode (1 ml/min) with helium 5.0 as the carrier gas and an oven program ranging from 60°C (for 1 min) to 260°C (15°C/min) to 280°C (1°C/min) to 320°C (25°C/min and hold for 5 min). The temperature of the transfer line connecting to the mass spectrometer was set to 295°C. The injector was a cold injection system (Gerstel, Mülheim, Germany) operated with a temperature program starting at 45°C and ramping to 300°C at a rate of 12°C/s.

*(d) Metabolite quantification.* Metabolites were quantified using MassHunter quantitative analysis software 7.0 (Agilent Technologies, Santa Clara, CA, USA). Untreated skin models or cell culture media were used as blanks. A matrix calibration was performed using skin models or culture medium spiked with defined amounts of the B[*a*]P metabolites under consideration (Table 1). The calibration series was prepared by dilution of stock solutions in DMSO, so that the DMSO concentration in the final solution did not exceed 0.01%. The concentration range of the standards varied depending on their limit of detection (Table 2). Calibration curves were checked for linearity and used for quantification of the analytes (Fig. S3B). The B[*a*]P-diones contained in the samples are present in solution in a redox equilibrium with their respective dihydroxy-B[*a*]P counterparts (55). During the derivatization reaction, B[*a*]P-diones are reduced to their corresponding dihydroxy-B[*a*]Ps, which are silylated and subsequently analyzed by GC-MS. Therefore, the ratio between diones and dihydroxy analogs cannot be determined by this method (Fig. S3A). In bacterial metabolism, B[*a*]P-dihydrodiols can be converted to dihydroxy-B[*a*]P by dihydrodiol dehydrogenases (56, 57). However, since beside the B[*a*]P-7,8-dihydrodiol and the B[*a*]P-9,10-dihydrodiol no other metabolites with the corresponding mass spectra (*m/z* = 430, *m/z* = 340) were measured in our experiments, the presence of B[*a*]P-diones is much more likely here. Given a similar concentration of the B[*a*]P-7,8-dihydrodiol in the uncolonized controls and the colonized skin models, but a significantly higher concentration of B[*a*]P-7-8-dione in the latter, the presence of the dione can also be assumed.

**TABLE 2:** External standards^*a*^

Substance	RT (min)	Quantifier *m/z*	Qualifier *m/z*	Allocation to IS^*b*^	Calibration range (pmol)
B[*a*]P	26.7	252	253	1	2.5–2,500
3-OH-B[*a*]P	34.9	340	341	2	3.7–1,850
7-OH-B[*a*]P	34,6	340	341	2	3.7–370
9-OH-B[*a*]P	33.7	340	341	2	3.7–370
B[*a*]P-1,6-dione	41.8	428	429	3	8.9–1780
B[*a*]P-6,12-dione	38.5	428	429	3	8.9–1,780
B[*a*]P-7,8-dione	42.2	428	429	3	8.9–1,780
B[*a*]P-7,10-dione	33.7	428	429	3	8.9–1,780
B[*a*]P-7,8-dihydrodiol	35.4	430	340	4	17.6–1,760
B[*a*]P-9,10-dihydrodiol	28.3	430	340	4	17.6–1,760
B[*a*]P-7,8,9,10-tetrahydrotetrol	36.7	404	191	5	18.7–1,870

a External standards used for identification and quantification of the B[*a*]P metabolites using GC-MS.

b Designation corresponding to the IS listed in Table 1.

### BPDE-DNA adduct quantification.

The BPDE-DNA adduct enzyme-linked immunosorbent assay (ELISA) (STA-357; Cell Biolabs, Inc., San Diego, CA, USA) was performed according to the manufacturer’s instructions to compare BPDE-DNA adduct formation in colonized skin and control tissues. Initially, the DNA of the skin models was isolated with a TRIzol-based protocol using the TRIzol reagent (Thermo Scientific, Waltham, MA, USA) as described earlier (58). The DNA concentration was determined using the Qubit dsDNA HS assay kit (Q32851; Thermo Scientific, Waltham, MA, USA) in the Qubit 2.0 fluorometer (Thermo Scientific, Waltham, MA, USA). The absorbance was measured at 450 nm using a BioTek Synergy Neo2 spectrophotometer (BioTek Instruments, Winooski, VT, USA).

### Statistical analysis.

All experiments were performed with at least three biological replicates if not stated otherwise. Data are presented as means and standard deviations (SD). GraphPad Prism 8 (Statcon, Witzenhausen, Germany) was used for data analysis, illustration, and statistical data processing, with analyses of multiple groups by one-way analysis of variance (ANOVA) with Bonferroni’s multiple-comparison test or ordinary two-way ANOVA being performed as appropriate. Data from the analytical investigations were normalized by dividing each value of the microbially colonized skin treated with B[*a*]P by the mean value of the respective control tissues treated with the same B[*a*]P stock solution but lacking skin bacteria and multiplying by 100 for the specification in percentages. For the evaluation of the Western blot data, the normalization was done by dividing the measurement of all different conditions by the value of the untreated control (no B[*a*]P and no bacteria) starting with the loading control (GAPDH) and then with the respective target. The normalization of data for immunofluorescence measurements was performed on the respective untreated control (no B[*a*]P and no bacteria), which was stained at the same time as the treatments. For the evaluation of the transcriptome data, the batch effect was included in the case of the B[*a*]P activating genes for comparability with our previous data (31). The batch effect considers the cultures treated with the same B[*a*]P stock solution. In general, a value of *P* of ≤0.05 was accepted as statistically significant.

### Data availability.

Raw and processed data files have been deposited in the Gene Expression Omnibus (GEO) data repository under accession number GSE171720.
