# Supplementary figures and images for: Commensal-Related Changes in the Epidermal Barrier Function Lead to Alterations in the Benzo[a]Pyrene Metabolite Profile and Its Distribution in 3D Skin
Source: mBio. 2021 Sep 28;12(5):e01223-21. doi: 10.1128/mBio.01223-21 (PMC8546866; doi:10.1128/mBio.01223-21)

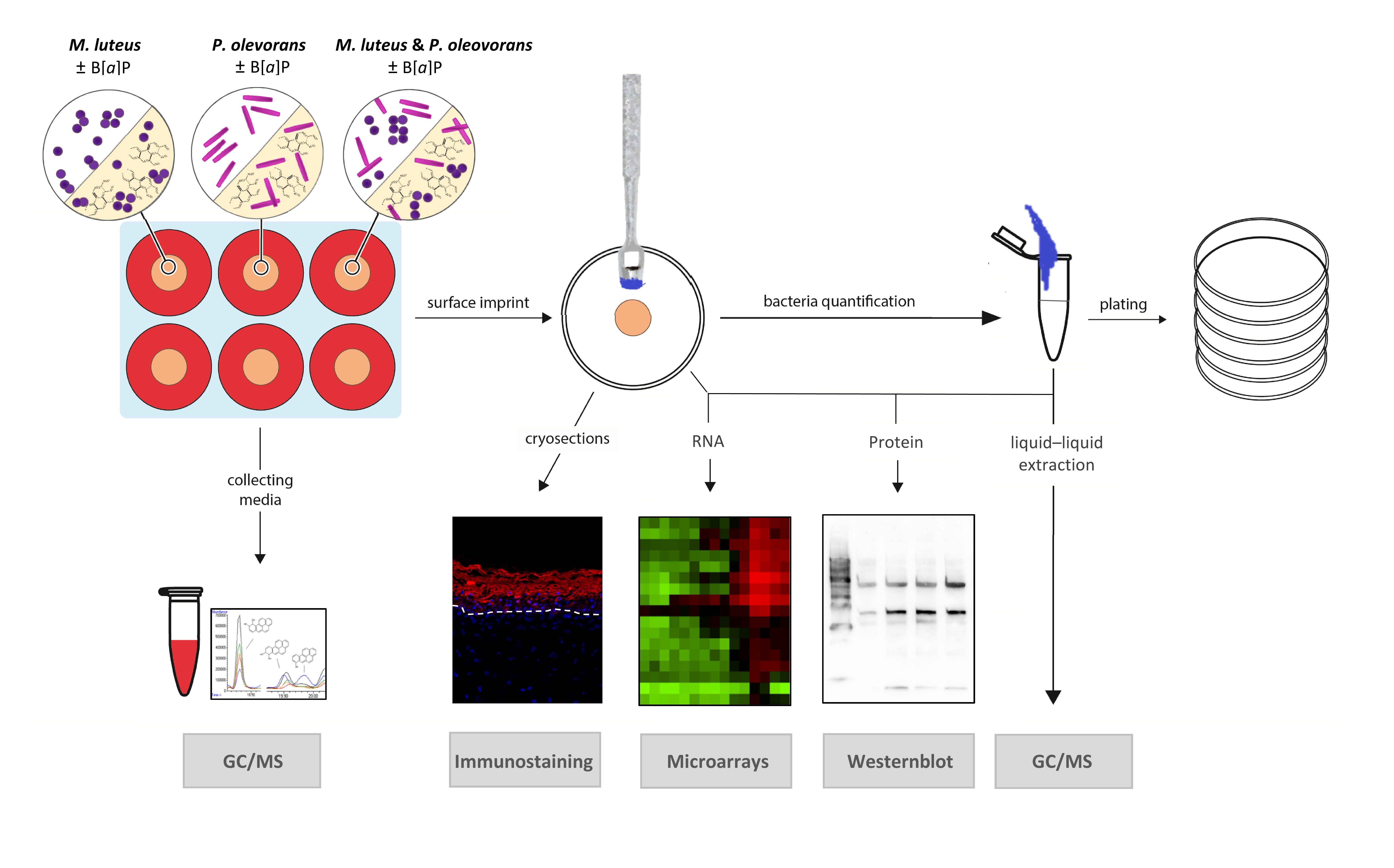

Supplement: DATA SET S1 [file mbio.01223-21-sd001.jpg]

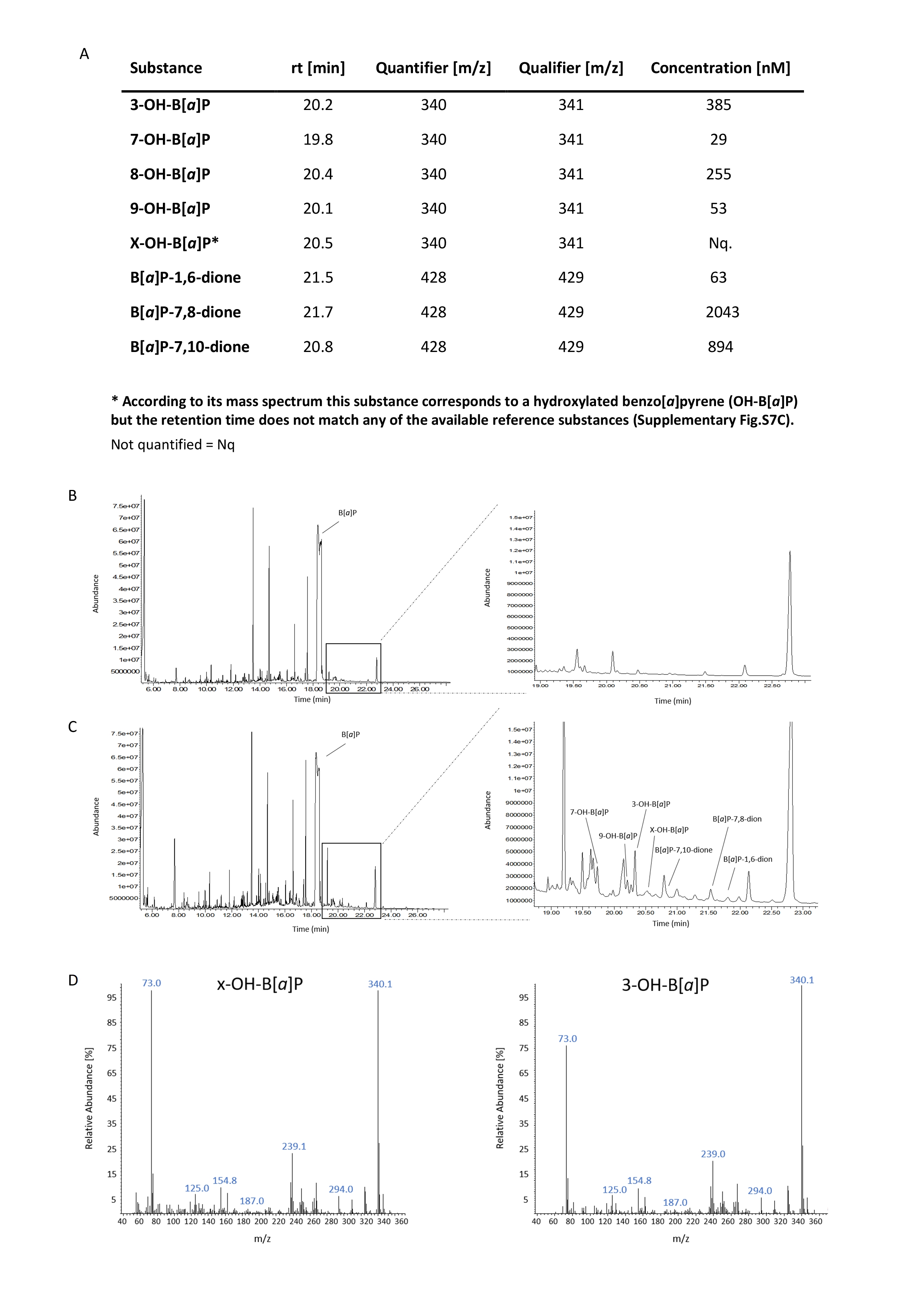

Supplement: FIG S4 [file mbio.01223-21-sf004.jpg]

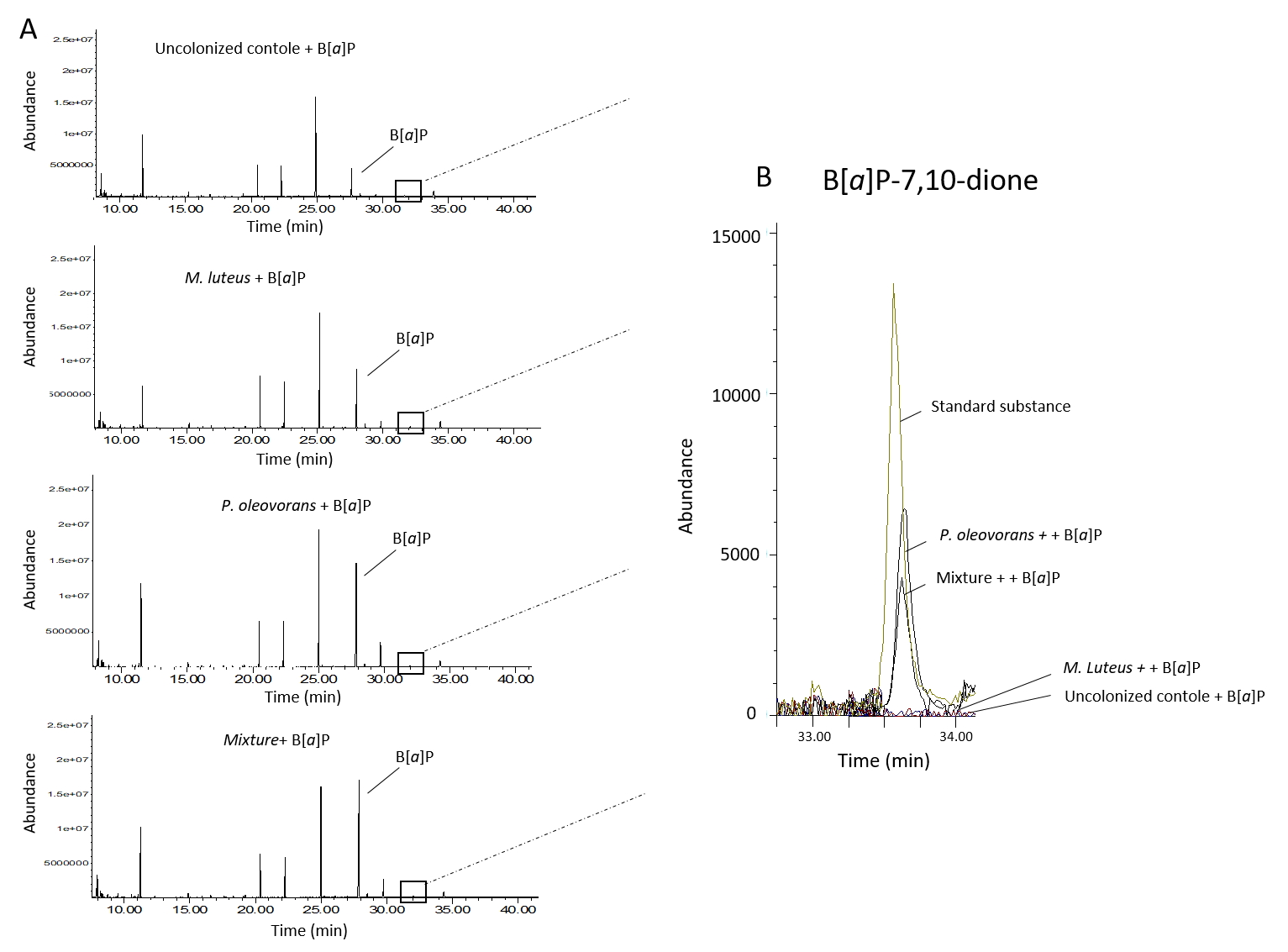

Supplement: FIG S5 [file mbio.01223-21-sf005.jpg]

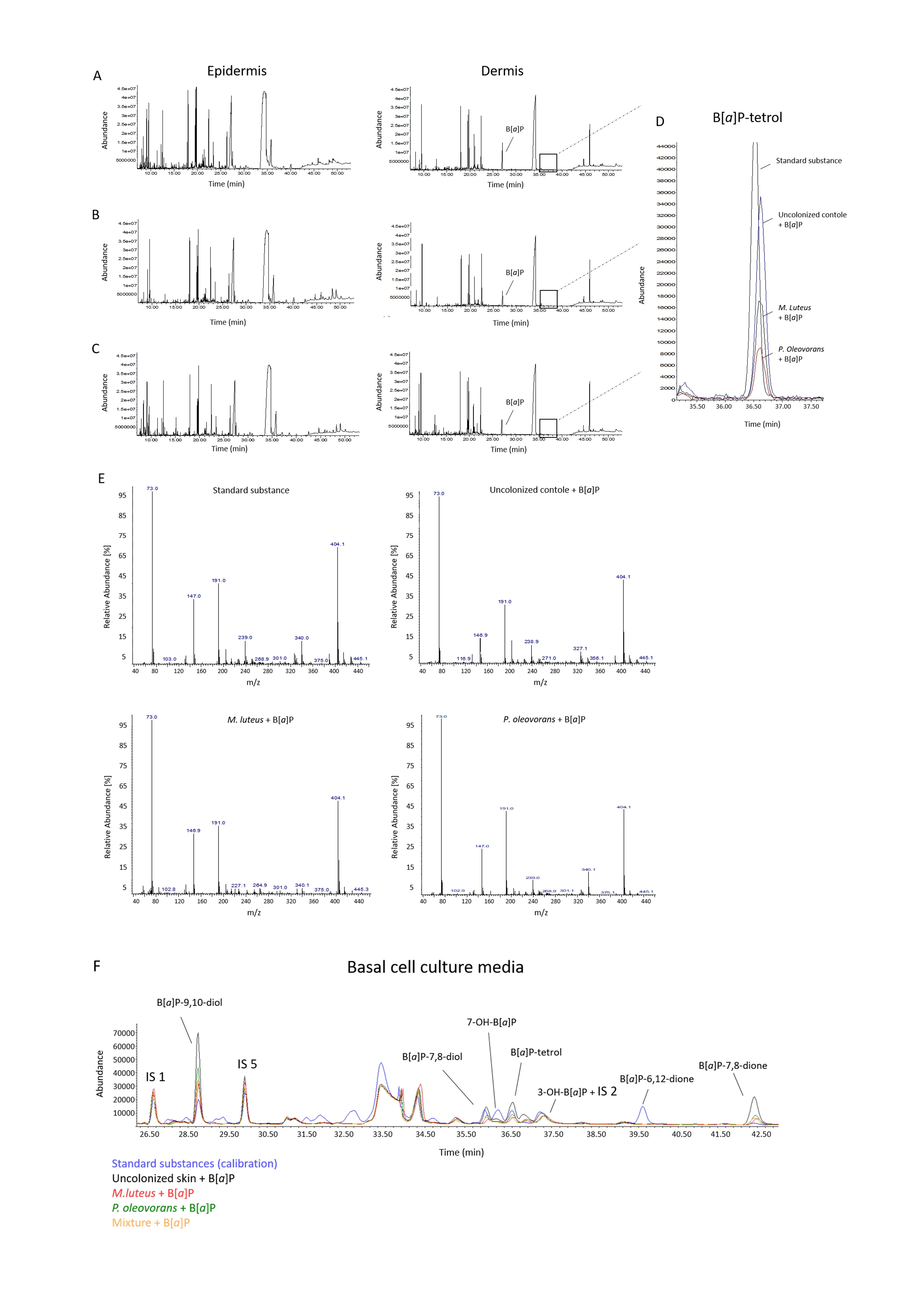

Supplement: FIG S6 [file mbio.01223-21-sf006.jpg]

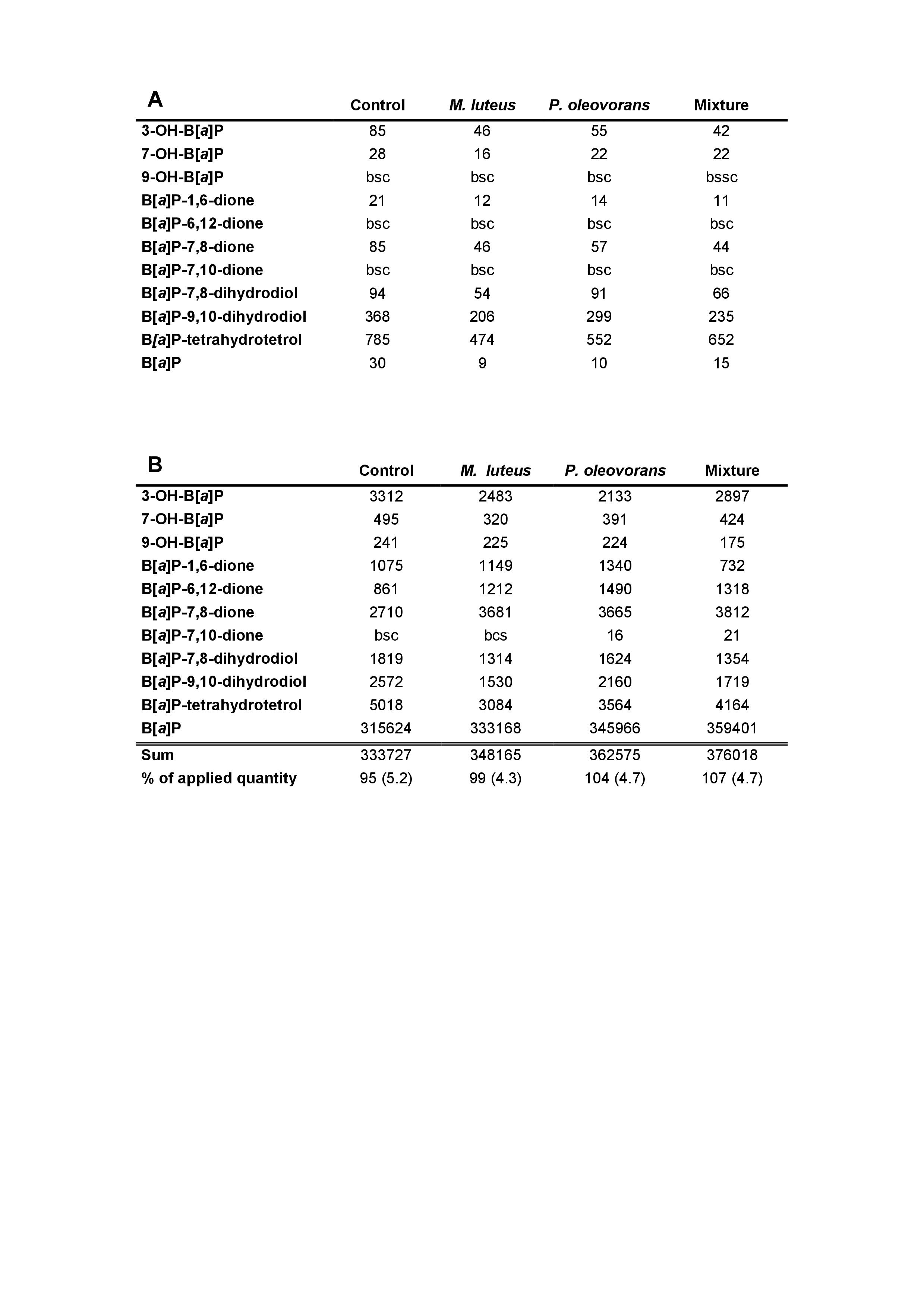

Supplement: TABLE S1 [file mbio.01223-21-st001.jpg]

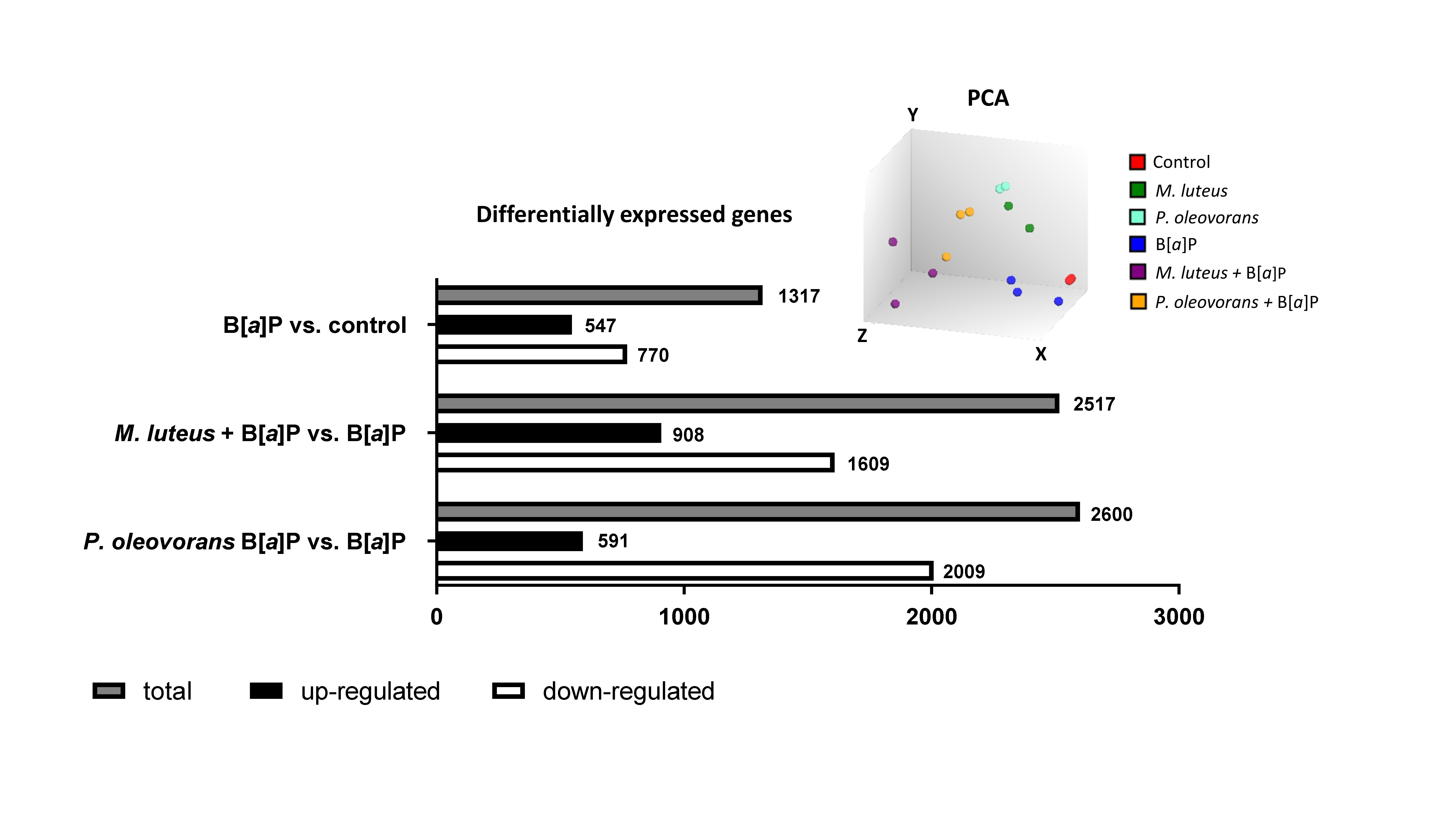

Supplement: FIG S7 [file mbio.01223-21-sf007.jpg]

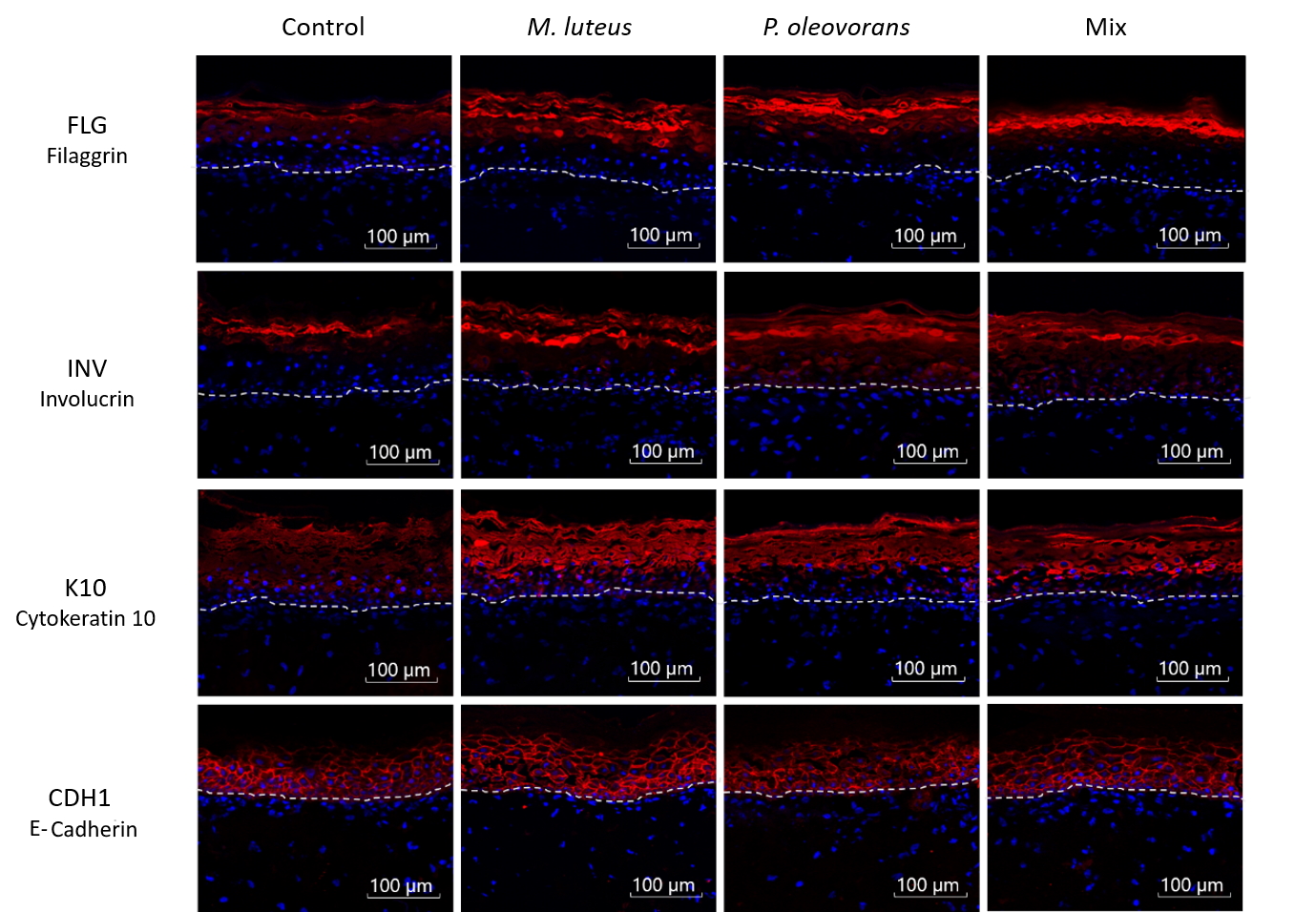

Supplement: FIG S8 [file mbio.01223-21-sf008.jpg]

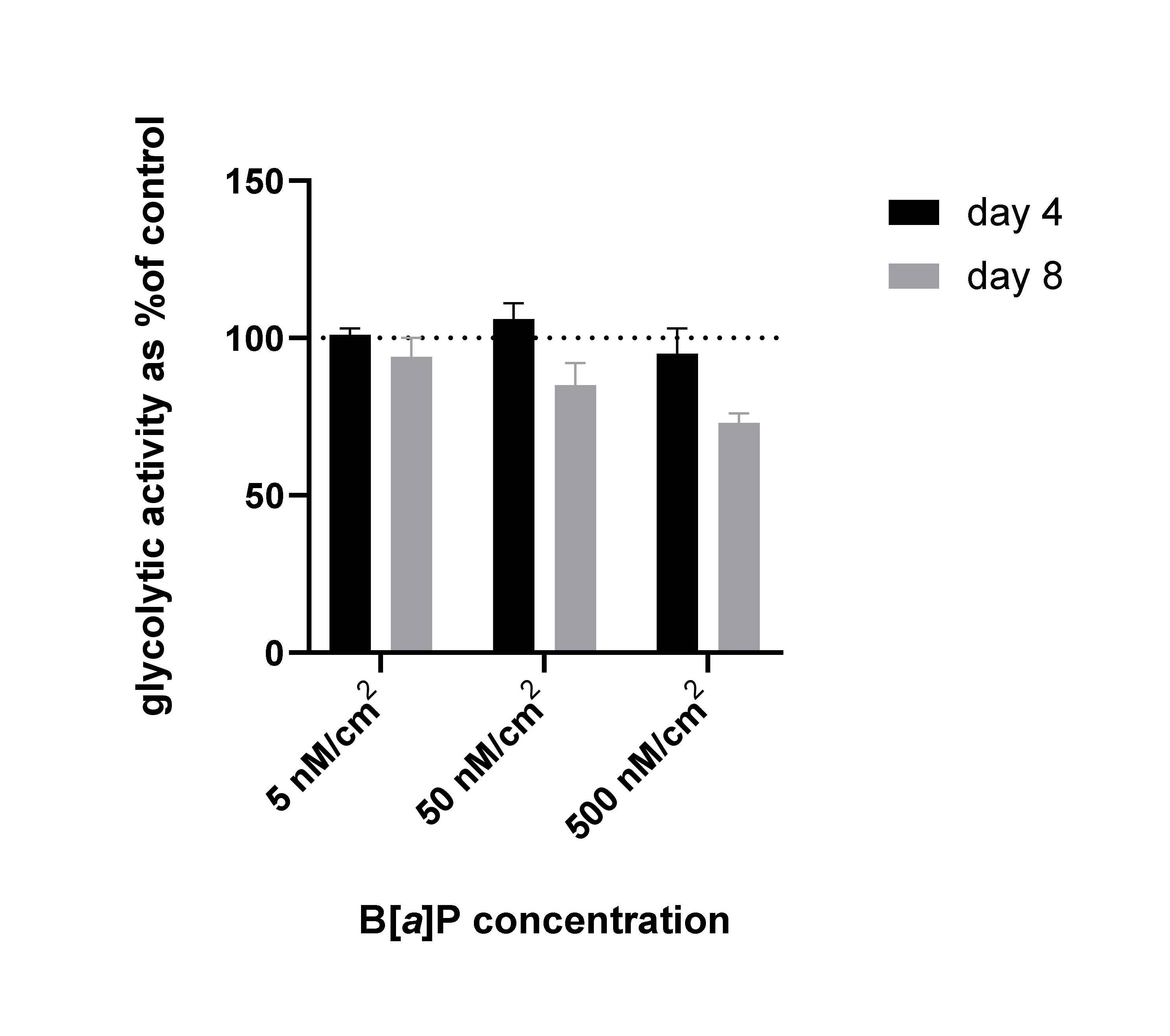

Supplement: FIG S1 [file mbio.01223-21-sf001.jpg]

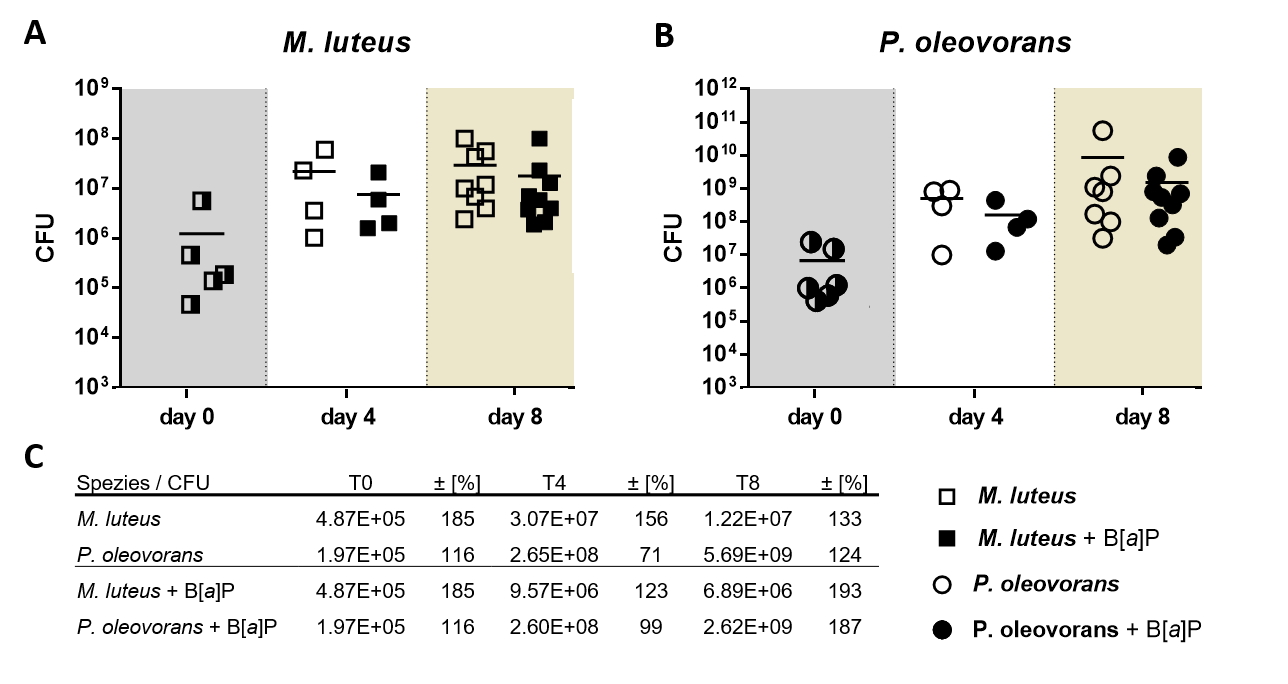

Supplement: FIG S2 [file mbio.01223-21-sf002.jpg]

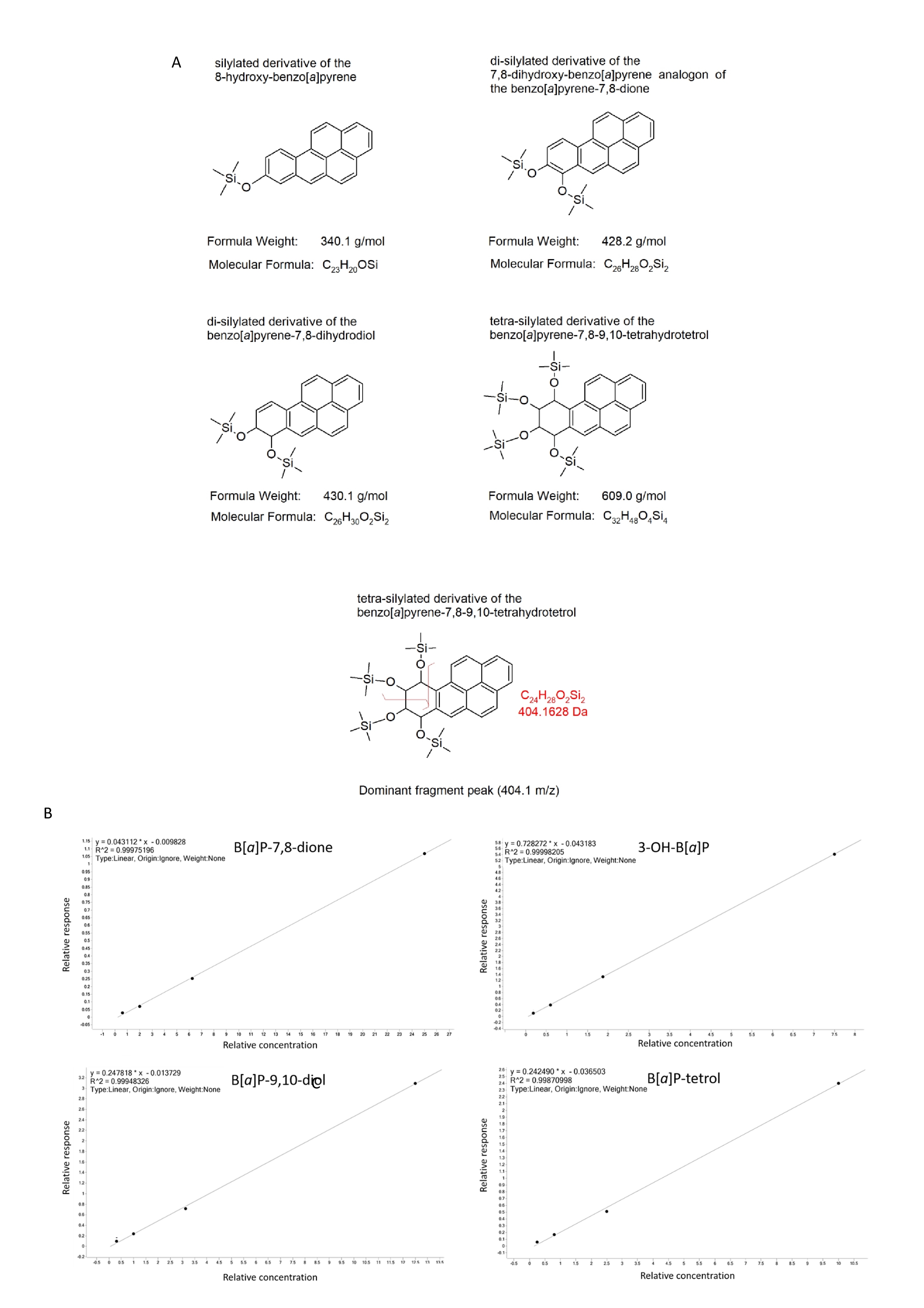

Supplement: FIG S3 [file mbio.01223-21-sf003.jpg]
